# Mapping Microbial Abundance and Prevalence to Changing Oxygen Concentration in Deep-Sea Sediments Using Machine Learning and Differential Abundance

**DOI:** 10.3389/fmicb.2022.804575

**Published:** 2022-05-18

**Authors:** Tor Einar Møller, Sven Le Moine Bauer, Bjarte Hannisdal, Rui Zhao, Tamara Baumberger, Desiree L. Roerdink, Amandine Dupuis, Ingunn H. Thorseth, Rolf Birger Pedersen, Steffen Leth Jørgensen

**Affiliations:** ^1^Department of Earth Science, University of Bergen, Bergen, Norway; ^2^Centre for Deep Sea Research, University of Bergen, Bergen, Norway; ^3^Bjerknes Centre for Climate Research, University of Bergen, Bergen, Norway; ^4^Department of Earth, Atmospheric, and Planetary Sciences, Massachusetts Institute of Technology, Cambridge, MA, United States; ^5^Cooperative Institute for Marine Ecosystem and Resources Studies, Oregon State University, Newport, OR, United States; ^6^IUT de Brest, Brest, France

**Keywords:** Support Vector Machines, Compositional Data Analysis, Arctic Mid-Ocean Ridge, Norwegian-Greenland Sea, threshold response, classification

## Abstract

Oxygen constitutes one of the strongest factors explaining microbial taxonomic variability in deep-sea sediments. However, deep-sea microbiome studies often lack the spatial resolution to study the oxygen gradient and transition zone beyond the oxic-anoxic dichotomy, thus leaving important questions regarding the microbial response to changing conditions unanswered. Here, we use machine learning and differential abundance analysis on 184 samples from 11 sediment cores retrieved along the Arctic Mid-Ocean Ridge to study how changing oxygen concentrations (1) are predicted by the relative abundance of higher taxa and (2) influence the distribution of individual Operational Taxonomic Units. We find that some of the most abundant classes of microorganisms can be used to classify samples according to oxygen concentration. At the level of Operational Taxonomic Units, however, representatives of common classes are not differentially abundant from high-oxic to low-oxic conditions. This weakened response to changing oxygen concentration suggests that the abundance and prevalence of highly abundant OTUs may be better explained by other variables than oxygen. Our results suggest that a relatively homogeneous microbiome is recruited to the benthos, and that the microbiome then becomes more heterogeneous as oxygen drops below 25 μM. Our analytical approach takes into account the oft-ignored compositional nature of relative abundance data, and provides a framework for extracting biologically meaningful associations from datasets spanning multiple sedimentary cores.

## 1. Introduction

Oxygen has a profound influence on microbial life and its associated activity (Glud, 2008; Teske, 2012; Orsi, 2018; Hoshino et al., 2020). Its mere presence is lethal to some microbes, whereas for others the oxygen tension exerts a critical control on their metabolism by influencing enzyme kinetics (Lu and Imlay, 2021). To aerobes, oxygen is the most energetically favourable electron acceptor (Froelich et al., 1979; Orsi, 2018), but it has been shown that common benthic microbial lineages exhibit growth patterns consistent with highly varying acceptance and usage of oxygen (Coskun et al., 2019). To anaerobes, conversely, oxygen is toxic and many defence mechanisms have evolved since oxygen first became abundant on Earth around 2.5 billion years ago (Lu and Imlay, 2021). As such, the presence or absence of oxygen has a strong impact on microbial community composition and structure in any given environment (Zhao et al., 2019; Hoshino et al., 2020). However, the microbial response to the oxygen gradient and the transition zone between oxic and anoxic conditions is not well studied.

Deep-sea sediments constitute very stable environments with well-defined oxygen profiles and host a vast microbial biosphere (Kallmeyer et al., 2012; Parkes et al., 2014; D'Hondt et al., 2015; Jørgensen and Marshall, 2016; Orsi, 2018). These sediments are, therefore, ideal environments to study the effect of changing oxygen concentration on microbial communities. Numerous studies have already aimed at providing an overview of the microbial inventory of deep-sea sediments, both in terms of biomass (e.g., Kallmeyer et al. 2012; Parkes et al. 2014) and taxonomic diversity (e.g., Hoshino et al. 2020; Zinger et al. 2011; Hoshino and Inagaki 2019) by analysing marker genes such as the 16S rRNA gene. Coherent and sustained sampling efforts of both microbial and geochemical data, combined with cost-efficient high throughput sequencing, as well as increased computational power have resulted in recent studies using data from multiple sediment cores, improving the understanding of the spatial distribution of microbial communities, as well as the relationship with their immediate geochemical environment at both regional and global scales. Examples of such regional studies include hadal trenches in the West-Pacific (Peoples et al., 2019; Hiraoka et al., 2020), the Mid-Atlantic (Vuillemin et al., 2019, 2020), the South-Atlantic Polar Front (Varliero et al., 2019), the Dorado Outcrop in the East-Pacific (Zinke et al., 2018), the South China Sea (Zhang et al., 2021), and the Norwegian-Greenland Sea (Jørgensen et al., 2012; Zhao et al., 2020) to name but a few. On the global scale, a recent report by Hoshino et al. (2020) has resulted in a globally spanning overview of microbial diversity in varying marine sediments with hitherto unprecedented detail. Their findings are in line with other studies that suggest that organic carbon content and the presence or absence of oxygen are key factors for explaining variability within sedimentary microbial populations in subsurface sediment (Jørgensen and Marshall, 2016; Orsi, 2018).

Consistent and sustained sampling efforts notwithstanding, studying changes in microbial communities as a function of geochemical variability in sediments is still faced with a number of challenges, many of these are related to inadequate context data and low spatial sampling resolution, ultimately limiting the possibility of using advanced data mining strategies to draw unambiguous conclusions based on abundance and prevalence patterns. Given adequate data, a variety of machine learning techniques allow new ways of exploring microbial interactions and map microbial response to environmental variables, both of which are key components of sedimentary microbiome studies (Qu et al., 2019; Goodswen et al., 2021; Marcos-Zambrano et al., 2021). Moreover, the compositional nature of OTU tables renders common correlation methods invalid because these fail to consider the varying row sums of each sample (Aitchison, 1982; Lovell et al., 2015; Gloor et al., 2017; Luz Calle, 2019). Compositionally sound techniques per definition acknowledge the arbitrary row sums imposed by various stages of sequence processing (Gloor et al., 2017). The abundance of individual OTUs or taxa is consequently expressed as proportions instead of read counts, and changes in abundance with respect to other OTUs or context variables is referred to as differential abundance (Lovell et al., 2015; Erb and Notredame, 2016; Gloor et al., 2017; Quinn et al., 2017). However, neither machine learning nor differential abundance techniques are commonly applied in sedimentary microbiome studies.

Here, we use Support Vector Machines (SVM) and differential abundance in order to investigate the response of common microbial taxa to the depletion of oxygen and onset of anoxia in marine sediments. Our dataset comprises 184 sediment samples from 11 consistently sampled and sequenced sediment cores collected along the Arctic Mid-Ocean Ridge (AMOR) in the Norwegian-Greenland Sea between 2010 and 2017. The sediment samples follow the oxygen gradient from >150 μM to anoxia. In order to alleviate and overcome loss of sensitivity due to under-sampling and allow for statistically valid testing, we adhere to CoDA principles (Gloor et al., 2017; Luz Calle, 2019). We show broad-scale prevalence and abundance patterns of cosmopolitan taxa suggested to constitute a core seafloor microbiome (Zinger et al., 2011; Bienhold et al., 2016) and show that many microbial classes predict the oxygen concentration of sediment samples substantially above null accuracy, indicating a persistent, intimate connection with declining oxygen concentration down-core. Our findings also suggest that a relatively homogeneous microbiome is recruited to the benthos from the overlying waters and only becomes more heterogeneous as oxygen drops into the 10–25 μM interval. This localises the onset of the oxic-anoxic transition zone, one of several geochemical transition zones relevant to down-core community assembly processes (Orsi, 2018). By mapping differential abundance of individual OTUs along the oxygen gradient we find marked differences in how closely the most abundant microbial classes adhere to the oxygen concentration gradient. Despite their strong ability to classify samples according to oxygen concentration intervals, highly abundant representatives of classes like Nitrososphaeria are nevertheless not differentially abundant across the oxygen gradient, suggesting that other variables like nitrate are required to fully explain their abundance and prevalence patterns in sub-surface sediments.

## 2. Materials and Methods

### 2.1. Coring Locations and Sediment Sampling

**Figure 1:**
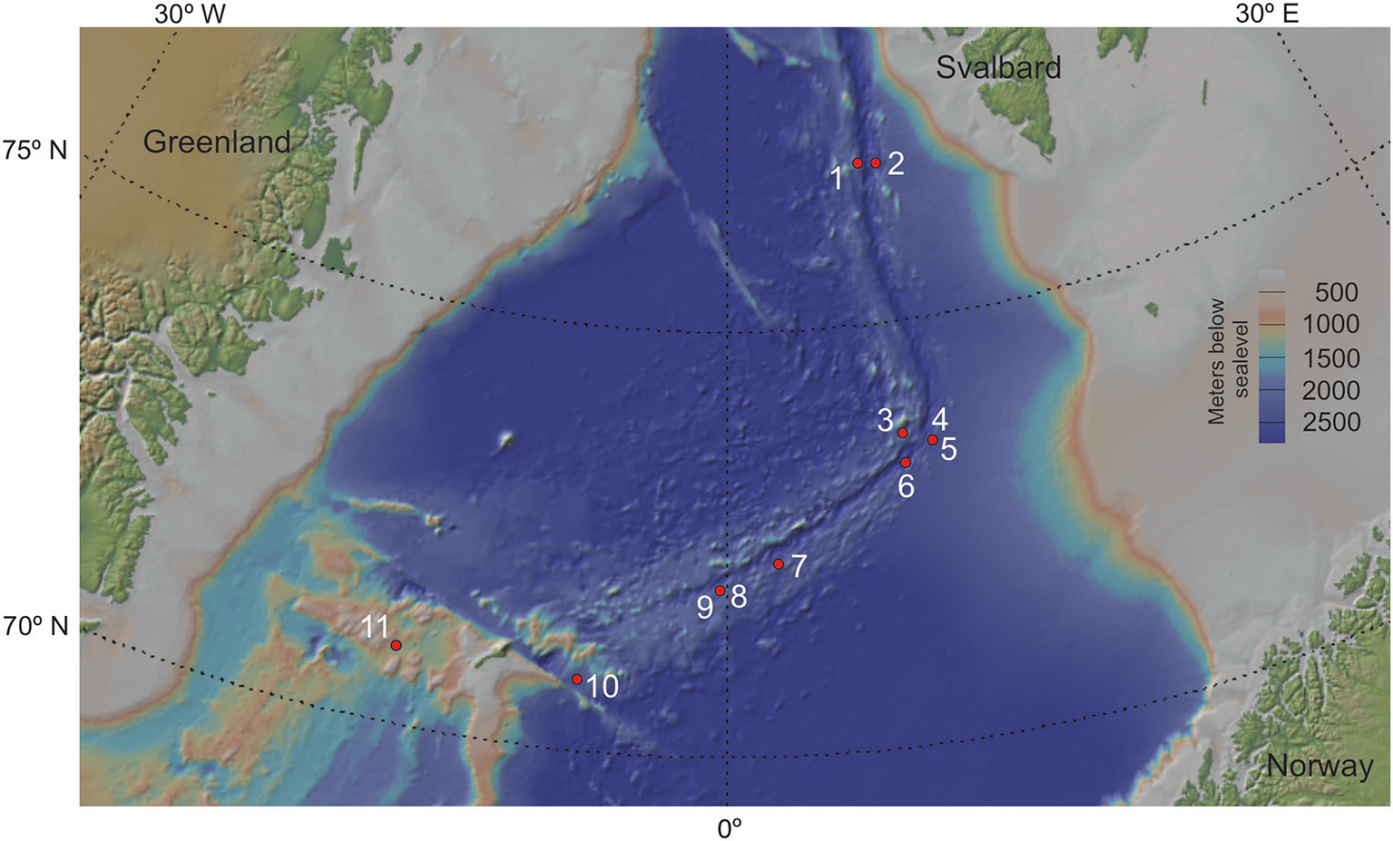
Coring locations for all sediment cores used in this study. Cores labelled in red were discarded during post-processing and decontamination. GS14_GC08 and GS17_GC04, as well as GS15_GC01 and GS17_GC02 were retrieved too close for their points to be discernible on this map. The map was created using GeoMapApp (v.3.6.10; https://geomapapp.org). Coring location for all 11 sediment cores used in the analyses of this study. Numbers from 1-11 refers to core name; 1: GS16_GC05, 2: GS16_GC06, 3: GS14_GC09, 4: GS15_GC01, 5: GS17_GC02, 6: GS14_GC12, 7: GS16_GC04, 8: GS14_GC08, 9: GS17_GC04, 10: GS17_GC05, 11: GS14_GC02. Note that site 4 and 5 are from approximately same location, so are 8 and 9. The map was created using GeoMapApp (v.3.6.10; https://geomapapp.org).

The initial dataset comprised data from 415 samples obtained from 16 individual sediment cores collected along the Arctic Mid-Ocean Ridge (AMOR) system from 2010 to 2017 during annual summer cruises to the Norwegian-Greenland Sea with R/V G.O. Sars. However, five cores were discarded due poor geochemical resolution, leaving 11 cores for downstream analyses (Figure 1). The sediment cores cover a geographic distance of 483 nautical miles from the Jan Mayen fracture zone in the south to the northern parts of the Knipovich ridge and span water depths from 1,036 to 3,493 meters below sea surface.

Onboard, cores were immediately split in halves and sub-samples for molecular microbial analyses were taken at regular depth intervals with sterile syringes from the middle section of each core (see the Supplementary Material for a complete sample overview). The samples were stored at −80°C until later onshore analyses.

Dissolved oxygen concentration was measured immediately after core-splitting using a needle-type fibre-optic oxygen microsensor connected to a MICROX TX3 single channel oxygen meter with a lower detection limit of 3.0 μM, which was calibrated according to the manufacturer's protocols (optodes, PreSens, Regensburg, Germany). Optodes were inserted manually into the sediments. For a complete overview of measurement depths, see Supplementary Material.

### 2.2. DNA Extraction, PCR Amplicon Sample Preparation and Sequencing

DNA extraction, sample preparation and sequencing for all samples follow the protocol previously described in (Zhao et al., 2020). In brief, following DNA extraction, DNA extracts were amplified in duplicate using the 519f (5′–CAGCMGCCGCGGTAA) forward and 805r (5′–GACTACHVGGGTATCTAATCC) reverse primers, covering the V4 of the prokaryotic 16S rRNA gene. Resulting amplicons were cleaned and pooled equimolarly before final sample preparation and sequencing using the Ion Torrent Personal Genome Machine (PGM) platform technology (Life Technologies). Sequencing was performed in eight separate batches. All batches were controlled for contamination using at least one extraction blank, and there were 15 blanks in total for the entire dataset (Supplementary Table S1).

### 2.3. Sequence Processing and Post-processing

Sequences were processed using VSEARCH (v.2.15.1; Rognes et al. 2016). First, Cutadapt (v.3.0; Martin 2011) was used to remove primers. Sequences were trimmed to 220 bp and then quality filtered allowing max 2 errors before dereplication. Sequences were then pooled, and dereplicated again before denovo and reference chimera detection. Following the first round of chimera detection, sequences were reverse-mapped to original fasta files before clustering at 97% similarity. Then followed another round of denovo and reference chimera detection. The full processing script is available along with all code necessary to reproduce the findings and results in this study (cf. Data Availability Statement). We assigned taxonomic classification to the identified OTUs using BLASTn (v.2.8.1; Zhang et al. 2000) and the LCA classifier from CREST (v.3; Lanzén et al. 2012), using version 128 of the Silva database (v.128; Quast et al. 2012) tailored towards environmental sequences.

In sequence post-processing, we first discarded all singletons before removing possible contaminants using decontam with negative control prevalence (v.1.6.0; Davis et al. 2018). All OTUs classified to genus level were then checked against a recently published list of laboratory contaminants (Eisenhofer et al., 2019). Any genus present on the list was removed from the dataset. Finally, GS14_GC14 was removed from the dataset after a preliminary visual inspection showed that samples from its sequencing run (CB3) clustered together rather than according to gradient variables, likely due to hydrothermal influence.

### 2.4. Sample Selection

Following post-processing and decontamination, we removed all sequenced samples with fewer than 1,000 reads. This threshold was set after inspecting plots of diversity (Shannon, Simpson) against sample size; diversity was considerably lower in samples with fewer than 1,000 reads, but there was no discernible pattern between the two in samples with more than 1,000 reads (Supplementary Figure S1). After discarding shallow-sequenced samples, we removed all OTUs represented with fewer than 100 reads. This threshold was set pragmatically to avoid problems related to inconsistent results when performing pairwise comparisons of very rare OTUs (see section 2.6.2 below concerning the use of the propd function). For example, an OTU could be assigned as a member of group A when comparing group A and B, B when comparing B and C, and C when comparing A and C.

Because the focus of this study is to investigate the influence of oxygen concentration and the transition into anoxic conditions on microbial communities, we retained the uppermost three samples from anoxic horizons in each core for analysis. The beta diversity exhibited by the anoxic community relative to the oxic community remained relatively constant when varying the number of anoxic samples per core (Supplementary Figure S2), allowing us to emphasise sample selection based on group size. Hence, the selected set of anoxic samples ensures that we capture the potential impact of anoxia while at the same time leaving the total number of anoxic samples comparable to subsets of oxic samples and thereby minimise group size bias. The final dataset consisted of 4,565,880 reads comprising 4,424 OTUs in 184 samples from 11 different sediment cores, with sampling horizons spanning from surface sediment to 190 cmbsf. The dataset contains cores where oxygen penetration depth varies between 10–116 cmbsf.

### 2.5. Categorisation of Oxic and Anoxic Samples

In order to analyse the oxygen gradient in-depth, we needed to acquire concentration values corresponding to each microbial sample. However, most microbial samples did not have exact depth-corresponding oxygen measurements. We, therefore, used non-linear regression fitted with a mathematical expression for exponential decay to interpolate oxygen concentration at each microbial sampling horizon; this method was more easily implemented and executed than other regression methods while providing comparable results (data not shown). Onboard oxygen measurements are usually discontinued once the measured concentration falls below detection limit. Extrapolation to microbial horizons in deeper layers, without oxygen measurements, led to noisy results with concentrations fluctuating between values above the oxygen sensor's detection limit (3 μM) to negative concentrations where zeros were expected. Any sample with an interpolated concentration <5 μM was, therefore, set to 0. The resulting profiles may be seen on Supplementary Figure S3.

We divided our dataset into seven different categories, each spanning different oxygen intervals based on interpolated oxygen concentration (Table 1). We are not aware of any biologically justified division of sediment samples based on oxygen concentration beyond the simple oxic-anoxic dichotomy and note that microbial response to changing oxygen concentration may differ between lineages, is not necessarily linear and in general is not well constrained. The division is therefore based on a pragmatic solution in which we let each category represent samples spanning a given oxygen interval such that group sizes are as even as possible in down-stream statistical analyses. Specifically, samples belonging to each of the seven categories were grouped to allow binary (two groups: high-/mid-oxic vs. low-/anoxic), ternary (three groups: high-/mid-oxic vs. low-oxic vs. anoxic) and quaternary (four groups: high-oxic vs. mid-oxic vs. low-oxic vs. anoxic) classification (Table 1).

**Table 1:** Category assignment, number of samples, corresponding oxygen concentration intervals, and grouping used in binary, ternary, and quaternary classification analyses.

			**Grouping**		
**Category**	**No. of samples**	**O**_**2**_ **[μM]**	**Binary**	**Ternary**	**Quaternary**
1	9	>150	High-/mid-oxic	High-/mid-oxic	High-oxic
2	10	150–100			
3	16	100–50			
4	19	50–25			
5	45	25–10			Mid-oxic
6	52	10–5	Low-/anoxic	Low-oxic	Low-oxic
7	32	<5		Anoxic	Anoxic

### 2.6. Statistical Analyses

OTU tables generated from sequencing data are compositional, meaning that their row sums are arbitrarily imposed by various stages of sample preparation and sequence processing (Gloor et al., 2017; Luz Calle, 2019). Techniques based on principles of Compositional Data Analysis (CoDA) incorporate statistical tools that deal with this problem without resorting to the controversial yet commonplace practice of rarefying samples to the same sequencing depth (McMurdie and Holmes, 2014). Moreover, CoDA principles discourage use of direct correlation due to negative correlation bias and consequently increased risk of spurious correlations in compositional data, particularly within data subsets (van den Boogaart and Tolosana-Delgado, 2013). Instead, they advocate analysing log-ratio transformed data, which both sidesteps the need for normalisation and relieves the compositional nature of the data (Erb and Notredame, 2016). Log-ratio transformations are sub-compositionally coherent, meaning that analysing subsets of the transformed data will not affect the relative distances within the analysed subset; permutation-invariance furthermore guarantees that the order of the samples is irrelevant to analysis (Luz Calle, 2019). These features make transformed data preferable to normalised data for investigating abundance and prevalence patterns of specific taxa, which is a core feature of most quantitatively oriented microbiological studies.

All analyses were performed directly on collections of OTUs grouped according to phylum, class and order levels. However, we only report analyses on class level because the order level had a relatively high fraction of unassigned sequence reads (35.9%) which left much of the dataset unexamined (compared with 6.3% unassigned sequences on class level). Several of the most abundant classes are contained within the same phyla (e.g., Proteobacteria) but possess very differing metabolisms, which prompted us to focus on class rather than phylum level to best capture these differences. Correlations are reported using Spearman's ρ.

#### 2.6.1. Classification Using Support Vector Machines

**Figure 2:**
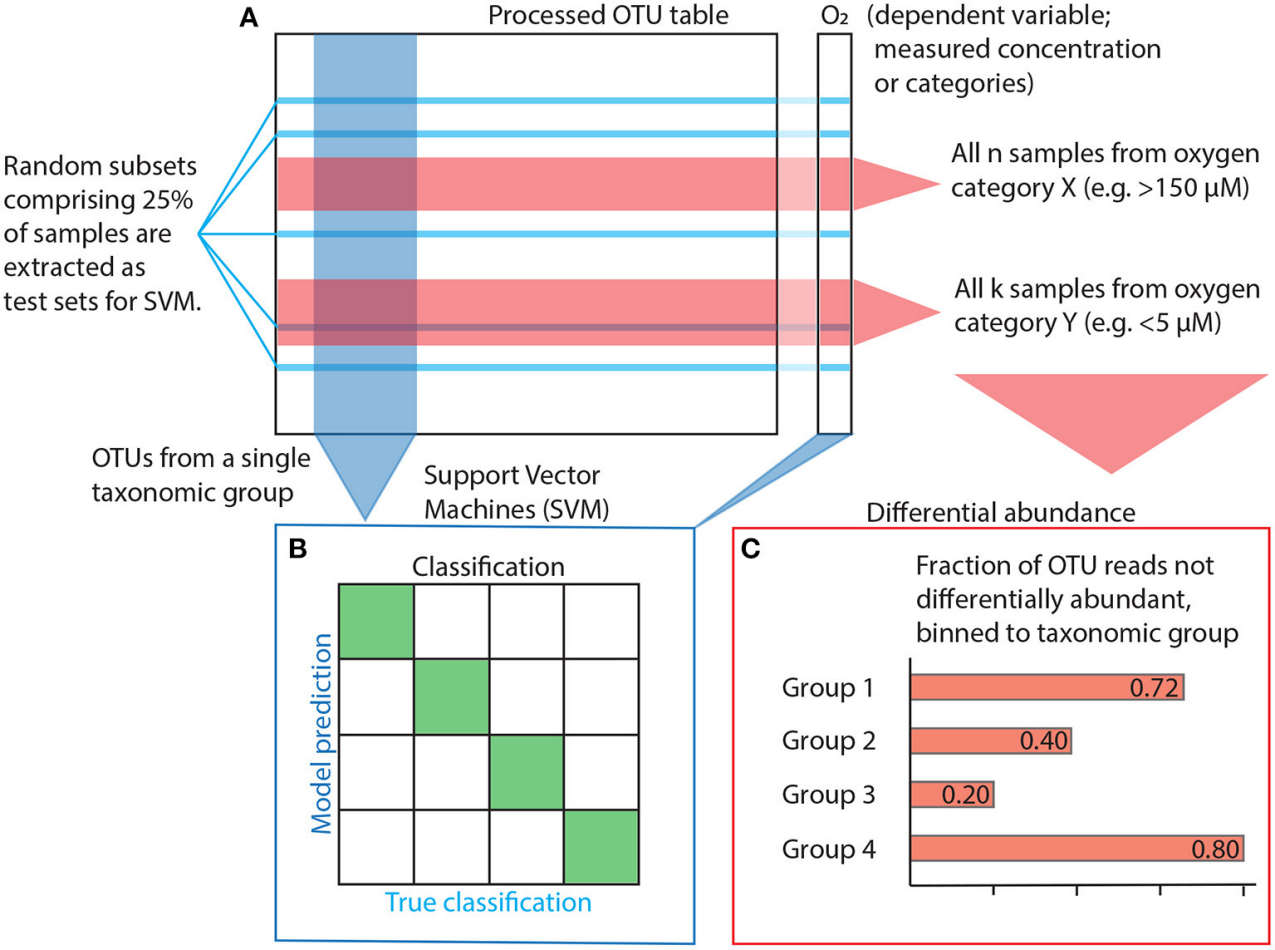
Statistical workflow. **(A)** The OTU table is subset and prepared depending on the method used. Subsetting for SVM is shown in blue, subsetting for differential abundance in red. **(B)** Model input for SVM is constructed using OTUs from within a single taxonomic group plus oxygen as the dependent factor variable for estimation of accuracy, i.e., the ability to classify a sample correctly. **(C)** Differential abundance is quantified as the variance of the log ratio (VLR) between all OTU pairs and across all oxygen category pairs (e.g., cats. 1 vs. 7, cf. Table 1). For a given pair of OTUs, if their VLR is low within categories but high between, then at least one of them is differentially abundant with regards to the other along the oxygen gradient spanned by the two categories. However, instead of looking at the differential abundance of OTUs, we focus on OTUs that are never differentially abundant, indicating that their variance does not change sufficiently in relation to any other OTU across the category pairs of interest. A high fraction non-differentially abundant reads suggests a weak response to changes in oxygen concentration.

One can assume that a tight, direct link between any taxonomic group and the oxygen gradient will be reflected in the group's ability to classify samples according to their oxygen concentration above random chance. Classification accuracy was determined using Support Vector Machines (SVM) (Cortes and Vapnik, 1995). SVM is a supervised, training-based method that uses distance functions (aka kernels; similar to distance functions in ordination analyses) to maximise the difference between factor levels (classification). Its computational speed and versatility makes it a suitable method for this study. Each analysis was run *n* = 128 iterations, and then another *n* = 128 iterations with the dependent variable randomly shuffled to obtain a comparable null result. Each iteration used a different, random subset comprising 25% of the samples for testing (Figure 2A).

All classification analyses were performed using a Radial Basis Function (RBF) kernel made for multi-factor classification (i.e., classification problems with more than two possible outcomes). Optimization parameters *C* and γ were determined using ten-fold cross-validation. Initial testing revealed sensitivity to uneven group sizes in the response variable, so we only performed analyses on assemblages of oxygen categories without a dominant (defined as comprising >60% of all samples) group. Accuracy was consequently calculated on oxygen categories grouped according to the three different setups listed in Table 1 to allow binary, ternary and quaternary classification (Figure 2B). In order to address the model's sensitivity to overfitting (i.e., redundancy among independent variables; Hawkins 2004), we ordered OTUs within each class by decreasing read count and created models for the first 10 OTUs before increasing numbers in increments of 20 until all OTUs were used.

#### 2.6.2. Differential OTU Abundance

We used differential abundance to quantify the extent to which individual OTUs respond to the oxygen gradient in AMOR sediment. We made pairwise comparisons of all OTUs and oxygen concentration categories (cf. Table 1). The differential abundance function requires non-zero data input, so prior to analysis, we applied zero-imputation. The differential abundance method uses the Variances of Log-Ratios (VLR) of individual OTU abundances to compare all OTU pairs in the dataset. The method checks whether the VLR is larger between than within two subsets of samples corresponding to some binary condition, e.g., oxic or anoxic conditions. If the variance is larger between than within each subset, at least one OTU is differentially abundant compared to the other with respect to the condition in question. We let the passing criterion for differential abundance be a disjointed proportionality (θ_*d*_) less than 0.50, meaning that the between-group VLR was at least twice as large as within-group VLR for the OTU pair in question (Erb et al., 2017). After removing pairs with a calculated false discovery rate >0.0005, we then retained only the 25% feature pairs with the lowest disjointed proportionality within each category. OTUs that were never present in any pair were then binned to higher taxonomic level (Figure 2C).

#### 2.6.3. Analysis Tools and Packages

All post-processing, statistical treatment and analysis was performed in the R statistical programming environment (v.3.6.3; R Core Team 2020) using the RStudio environment (v.1.1.463; RStudio Team 2016). Plots were generated using the ggplot2 (v.3.3.3; Wickham 2011) and egg (v.0.4.5; Auguie 2019) packages. Zero-imputation was applied using a slightly modified in-house version of the cmultRepl function in the package zCompositions (v.1.3.3.1; Palarea-Albaladejo and Martin-Fernandez 2015). Centred log-ratio transformation was done using the clr function in the package compositions (v.2.01; van den Boogaart et al. 2021). Classification was performed using kernlab (v.0.9.29, Karatzoglou et al. 2004) as wrapped by the caret package (v.6.0-85; Kuhn 2008). Differential abundance was calculated using the propd function from the proper package (v.4.2.6; Quinn et al. 2017).

## 3. Results and Discussion

### 3.1. Classification of Oxygen Concentration From OTU Abundance and Prevalence Patterns

Among the 34 phyla, 189 classes, 196 orders and 355 families identified in our post-processed dataset, we find that across all investigated depths, the top ten abundant classes along the Arctic Mid-Ocean Ridge (AMOR) are Alphaproteobacteria (18.0%), Nitrososphaeria (11.2%), the S085 (10.1%), Planctomycetacia (9.0%), Gammaproteobacteria (8.1%), Phycisphaerae (4.5%), Deltaproteobacteria (4.5%), MD2896-B214 (3.8%), Chloroflexi Subdivision 5 (SAR202) (2.9%), and Pacearchaeota (2.5%), reflecting global abundance patterns in deep-sea sediments (Orcutt et al., 2011; Zinger et al., 2011; Parkes et al., 2014; Zinke et al., 2018; Cui et al., 2019; Varliero et al., 2019; Vuillemin et al., 2019; Hiraoka et al., 2020; Hoshino et al., 2020; Schauberger et al., 2021). Their relative abundances, as well as those of all phyla and orders exceeding 1% relative abundance are shown on Supplementary Figures S4–S6.

**Figure 3:**
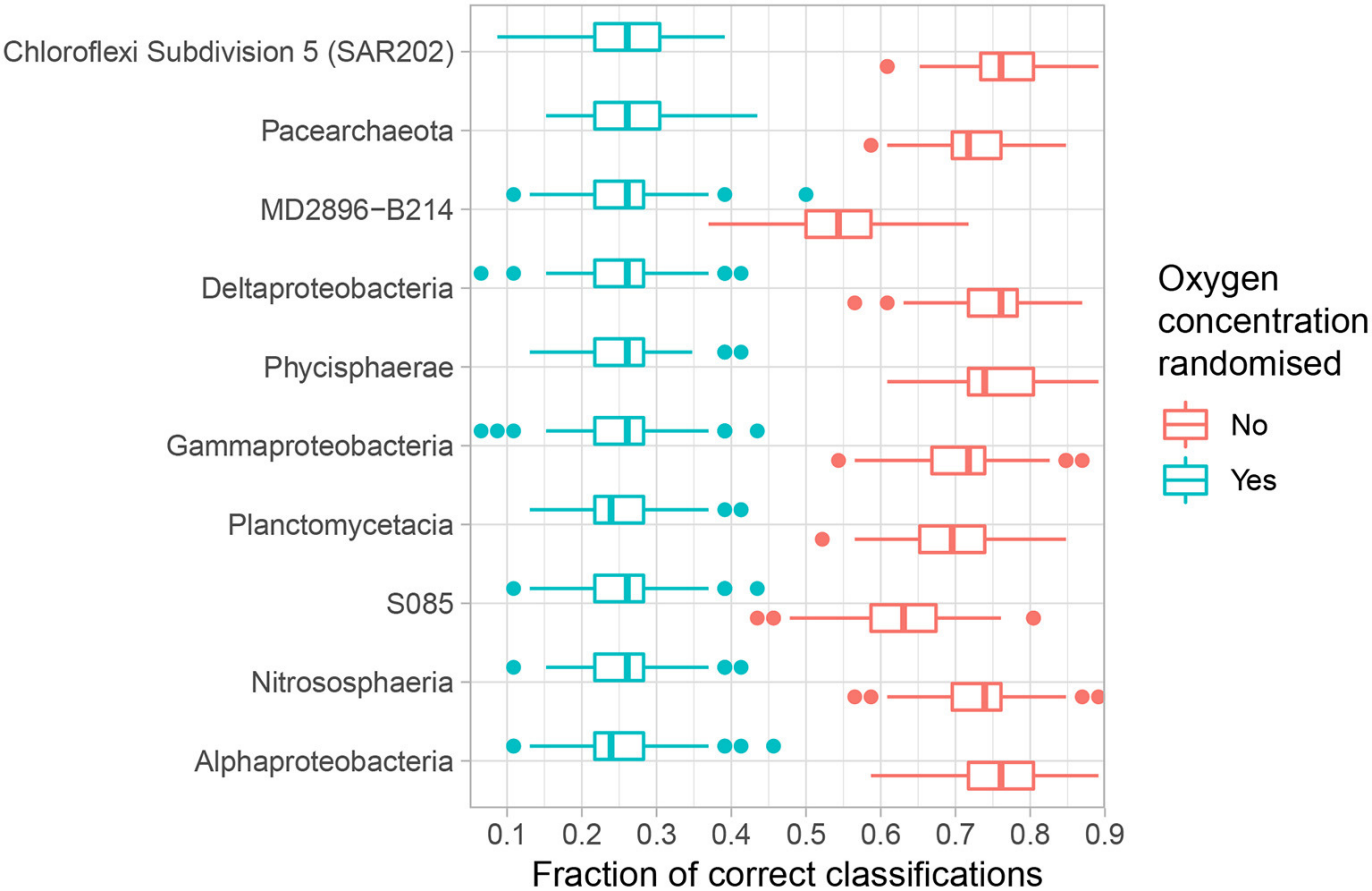
Accuracy of the top 10 abundant classes in quaternary classification analysis (Table 1). Analyses were repeated *n* = 128 times. Boxes denote the 25–75 percentiles, whiskers roughly the 95% confidence interval (Wickham, 2011). Blue boxes denote results where the dependent variable has been shuffled to signify results expected by random chance, red boxes with no randomisation. Relative abundance decreases upwards.

If the concentration of oxygen indeed influences the abundance and prevalence patterns of the microbial community, and furthermore specific classes, then we should be able to use said patterns to predict oxygen concentration better than random chance. The speed, sensitivity and versatility of SVM makes it an appropriate tool to investigate if such predictions can be made. We focus on the 10 most abundant classes because the most common taxa are expected to exhibit the most sensitive response to key environmental variables (ter Steege et al., 2013; de Vargas et al., 2015; Hannisdal et al., 2017) and first measure how accurately the OTUs comprising each class classify oxygen concentration according to four intervals: high-oxic: >25 μM, mid-oxic: 10–25 μM, low-oxic 5–10 μM, and anoxic <5 μM (Table 1, Figure 3). Chloroflexi Subdivision 5 (SAR202), Alphaproteobacteria and Deltaproteobacteria all achieve median classification rates (the percentage of correctly assigned samples) above 76% (Supplementary Table S3). The lowest median classification rate for any investigated class is 54%, which is still significantly higher than the 26.1% expected by random chance (Figure 3).

**Figure 4:**
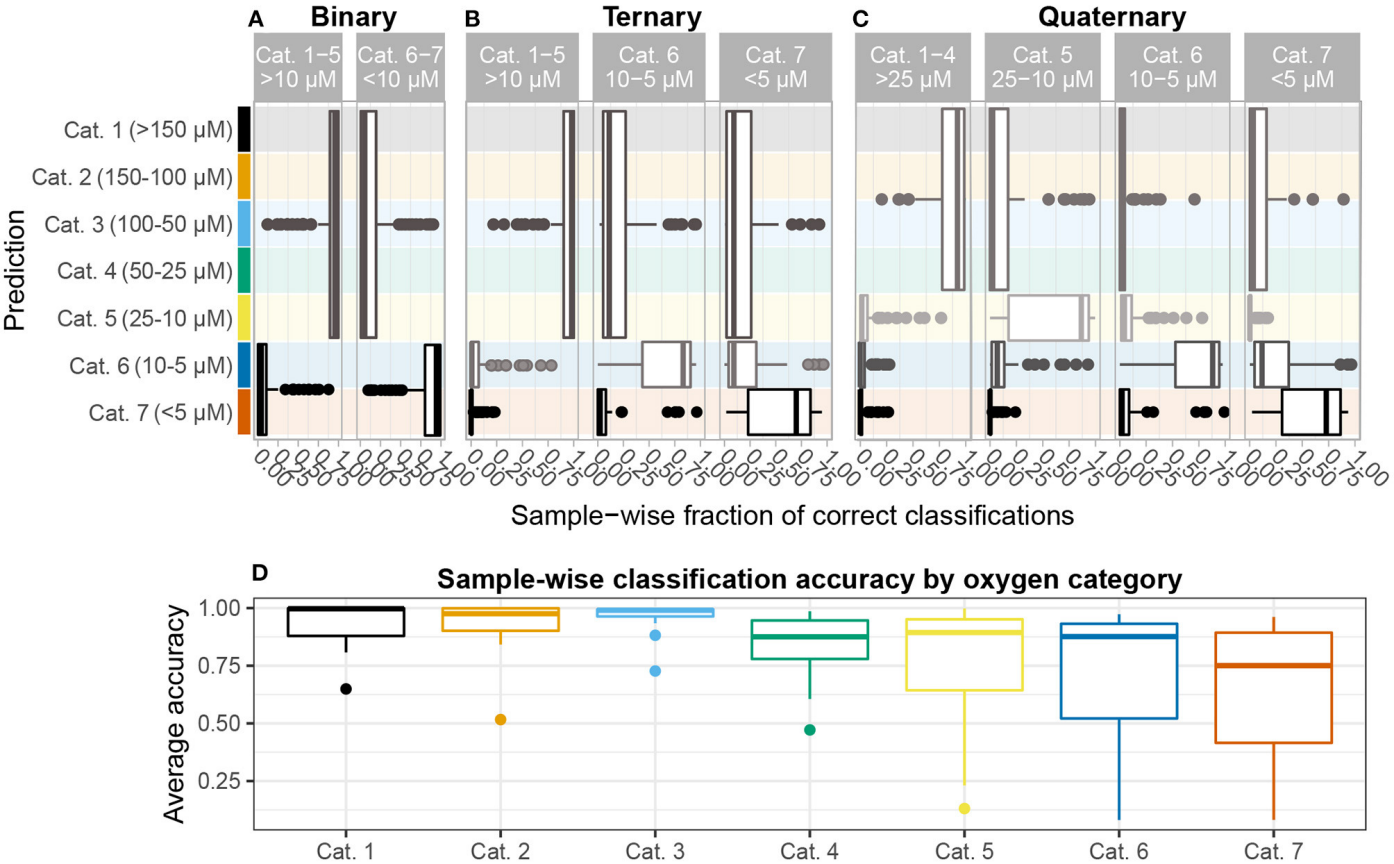
Sample-wise accuracy. All analyses report average accuracy over *n* = 128 iterations. Results are shown for **(A)** binary, **(B)** ternary, and **(C)** quaternary classification problems. The height of each box denotes the oxygen categories comprising each group in the classification problem. In the ternary and quaternary classification problems, median accuracy tends to decrease as oxygen depletes. **(D)** The average accuracy for each sample over all three classification problems shown on **(A–C)** are grouped by oxygen category. A higher fraction of samples with oxygen concentration >25μM, corresponding to categories 1–4, are classified correctly than 5–7, all of which suffer from a tail of seldom-correctly classified samples, indicating that communities inhabiting sediments with <25μM oxygen concentration are comparatively more heterogeneous and, therefore, harder to delineate statistically.

Between-core differences in oxygen penetration depth (10–116 cmbsf), as well as the much stronger correlation of per-sample classification accuracy with oxygen category (ρ=-0.43, p < 10^-9^) than with depth (ρ = −0.46, *p* = 0.00032), as well as direct comparison of classification rates using detrended OTU data (see Supplementary Methods for details), suggest that classification rates are not strongly confounded by sediment depth. Our results show that the composition of taxonomic groups common in AMOR sediments follow changes in oxygen concentration beyond the simple oxic-anoxic binary (Figure 4).

Optimal classification is typically achieved using only a fraction of the OTUs comprising each class. Adding additional OTUs after reaching the optimum, however, does not notably change classification rates (Supplementary Figure S7), a phenomenon known as overfitting (Hawkins, 2004). Median classification rate at the optimum is not significantly correlated with the relative abundance (Spearman, *p* = 0.99), suggesting that there is no relationship between relative abundance and classification rate for the top 10 abundant classes. Moreover, maximising the classification rate is not connected to the size of the fraction of OTUs within each class needed to do so (Spearman, *p* = 0.86). Thus, even though the conditions in the deep-sea benthos have been suggested to favour certain taxonomic groups over others and thus skewing the population seeded from the overlying waters (Walsh et al., 2016), the lack of a clear correlation between abundance and classification rates, unsurprisingly, suggests that oxygen alone cannot be invoked to explain this skew.

Due to uncertainty in the interpolated oxygen profiles, all values below 5 μM were set to 0 (cf. section 2.5). However, the detection limit for the oxygen measurement apparatus is 3 μM, so in order to test whether the fraction of misclassified anoxic samples could be explained by the selected threshold, we ran the analyses shown on Figure 3 again for 16 iterations on the same dataset changing only the threshold for labelling a sample as anoxic from 5 to 3 μM. Consequently, 10 samples were moved from category 7 (*n* = 32 to 22) to category 6 (*n* = 52 to 62). If the lowered threshold resulted in more “true” division of samples into categories, this should have been visible as a higher fraction of samples correctly classified as anoxic, but instead classification accuracy decreased considerably (Supplementary Figure S8).

Classification accuracy (how often a specific sediment sample is assigned correctly) is strongly correlated with oxygen category (ρ= −0.43, *p* < 10^-9^). Dividing samples according to the 7 categories introduced in Table 1, we find that mean accuracy decreases significantly from categories 1–4 to 5 and 6 and then to 7 (*p* < 3 × 10^-6^). While approximately 63% of the samples in categories 1–4 (>25 μM) are correctly classified at least 90% of the time, mean accuracy for anoxic (category 7) samples is 67% (Figure 4D). In order for the category 1–4 samples to achieve such high accuracy across multiple classes and sample grouping (i.e., binary, ternary, quaternary) the taxonomic composition of their microbial communities must be internally stable and relatively homogeneous. No specific core or coring location along AMOR stands out among this subset, indicating that recruitment from Norwegian Sea Deep Water, which overlies most of AMOR (Swift and Koltermann, 1988; Le Moine Bauer et al., 2018), is regionally homogeneous. A similar conclusion was drawn by Schauberger et al. (2021), who investigated Hadal trenches in the Pacific and found that community dissimilarity was better explained by geochemical gradients than by spatial distance between coring sites.

In ternary classification (high-/mid-oxic: >10 μM, low-oxic: 5–10 μM, anoxic: <5 μM), samples are disproportionally classified as high-/mid-oxic (Figure 4B). This contrasts with the quaternary grouping, where samples in the 10–25 μM interval, i.e., category 5, form a separate group, and these samples are disproportionally misclassified as low-oxic (5–10 μM) (Figure 4C). The change in classification patterns from ternary to quaternary grouping suggests that category 5 samples are taxonomically more heterogeneous than categories 1–4 (>25 μM) combined. Because oxygen penetration depth varies between cores (10–116 cmbsf), sediment depth or age cannot explain the observed changes. This finding, therefore, indicates that taxonomic changes associated with the transition to anoxia starts within this concentration interval, setting an empirically informed threshold for the first of several geochemical transition zones that shape community assembly in sub-surface sediment (Orsi, 2018; Zhao et al., 2019, 2020). It has previously been argued that the assembly of a core subsurface microbial community occurs at the transition between bioturbated and non-bioturbated zones (Chen et al., 2017b; Petro et al., 2019), and that ventilation brought about by bioturbation plays an important part in governing microbial community assembly and structure in bioturbated sediments (Deng et al., 2020). In light of our results, we propose that the initial assembly of a subsurface community starts somewhere between 10–25 μM oxygen concentration, irrespective of bioturbation intensity and depth.

**Figure 5:**
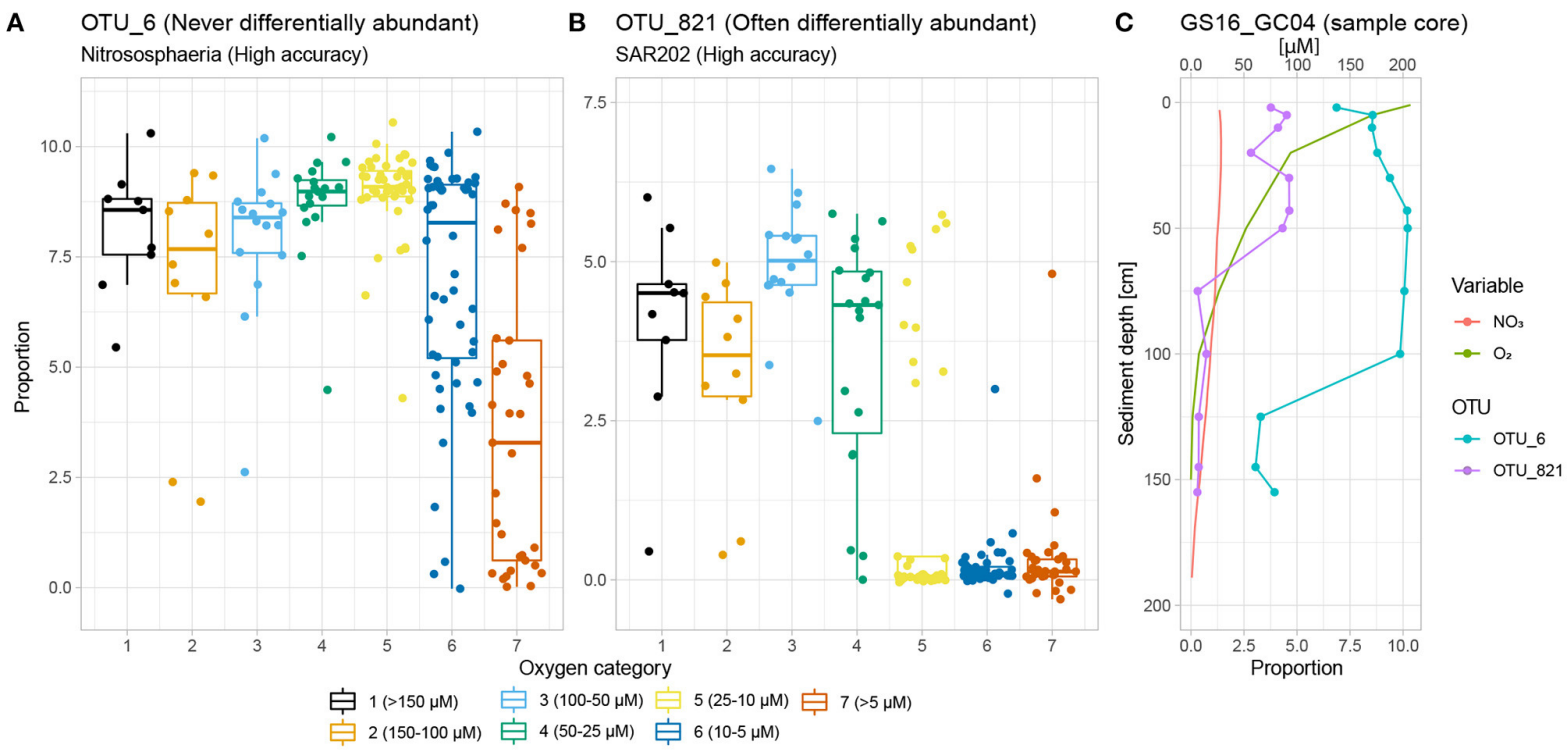
Classification rates vs. differential abundance. **(A)** OTU_6 belongs to Nitrososphaeria, which is a very strong SVM classifier. It is, however, never differentially abundant with regards to the oxygen gradient. This is because its large spread in relative abundance within categories 6 and 7 increases the within-group VLR against any other OTU (i.e., high θ_*d*_). **(B)** OTU_821 (SAR202, Chloroflexi), on the other hand, exhibits a threshold response to declining oxygen concentration and is clearly differentially abundant between categories 1 vs. 6 and 7 by the settings of this study. Within-group variability for categories 1 (high relative abundance), 6 and 7 (low relative abundance) are all low, indicating that OTU_821 will contribute to low within-group and high between-group VLR when paired with other OTUs (i.e., low θ_*d*_). **(C)** GS16_GC04 exemplifies the two OTUs' different behaviour with regards to the oxygen gradient, both of which nevertheless result in high classification rate with SVM.

Figures 5B,C exemplify how the abundance pattern of a representative of Chloroflexi Subdivision5 (SAR202) relates to the oxygen gradient in a way that would cause disproportionate classification as high-/mid-oxic in ternary classification but ambiguity between the mid-, low- and anoxic categories in quaternary classification. Our findings align with those of Chen et al. (2017a) who, following a 100-day incubation experiment at <20 μM oxygen concentration found that the microbial community, sampled from a tidal flat, was harbouring both anaerobic and aerobic metabolisms, showing that microbial communities do not abide strongly to the inferred redox profile in transition zones. As oxygen depletes and anoxia spreads, new micro-niches are established that enable growth of previously inhibited microbes by locally mobilising terminal electron acceptors like manganese and iron (Jørgensen, 1977; Coskun et al., 2019). Furthermore, highly sensitive oxygen measurements have recently resulted in the formation of a theoretical basis for aerobic growth in environments with nanomolar oxygen concentration (Zakem and Follows, 2017). Growth under these conditions have also been demonstrated in the laboratory (Stolper et al., 2010). A better understanding of the plethora of niches that arise in the transition from oxic to anoxic sediments might help better constrain the factors that limit or allow growth of uncultivable microbial lineages, but more targeted research is necessary to further pursue this topic.

Our results indicate that oxygen concentration is a very strong explanatory variable for the majority of common microbial classes present in subsurface sediments along AMOR. As such, our findings reflect, but further nuance, those of Hoshino et al. (2020), who show that the oxic-anoxic dichotomy correlates significantly with the microbial taxonomic composition in deep-sea sediments globally. The microbial classes most accurately classifying samples by oxygen concentration interval, such as Alphaproteobacteria and Nitrososphaeria (Figure 3), are also the most abundant classes in our samples, be it oxic or anoxic. Nevertheless, our results also show that there is no significant relationship between strong classification rate and relative abundance among the top 10 abundant classes in general. However, because our analyses do not extend to the rare biosphere, our results cannot be generalised to the microbial population as a whole. Based on broad empirical evidence, it has been suggested that the most abundant members of an ecosystem should also be the best predictors of key environmental variables (ter Steege et al., 2013; de Vargas et al., 2015; Hannisdal et al., 2017). Following this logic, a subset consisting of highly abundant OTUs, regardless of class, should outperform any individual class consisting of both abundant and rare OTUs. Classification rates for all OTUs exceeding 1% (*n* = 12), 0.3% (*n* = 53) and 0.1% (*n* = 118) relative abundance are nevertheless all lower than that achieved by Alphaproteobacteria (Supplementary Figure S9). This finding is surprising, and suggests that the rare members of abundant classes, which may exhibit narrow niche breadth for example in relation to oxygen concentration (Lynch and Neufeld, 2015), may play a key role in determining the overall ecological response to oxygen depletion on a higher taxonomic level. The relative inability of the most abundant OTUs to classify sediment samples according to oxygen concentration furthermore suggests that a wider suite of explanatory variables must be applied to fully contextualise their abundance and prevalence patterns. Previous studies have shown that community assembly in subsurface sediments is driven mainly by selective pressure favouring persisting lineages able to survive increasing energy limitation (Petro et al., 2017; Starnawski et al., 2017). Indeed, the strong dependency of available organic carbon on both initial cell counts, as well as the community structure in marine sediments has been repeatedly highlighted (e.g., Jørgensen and Marshall 2016; Starnawski et al. 2017; Deng et al. 2020). Furthermore, recent studies have shown in detail how the sources and composition of organic matter may influence community composition in relatively young sediments (Chen et al., 2017b; Deng et al., 2020). However, TOC profiles, available for the majority of our cores, do not exhibit a strong decline (Supplementary Figure S10), and only in three of 11 cores did organic carbon content correlate significantly with depth (Spearman, p < 0.05). This could be because our cores are too short (<4 m) for any such trend to be clear, or that the measured TOC at depth is no longer degradable and hence constitutes a refractory pool beyond access for the deeper microbial communities.

The results presented thus far show that even though overall abundance patterns exhibited by the most common classes are predictive of oxygen concentration, this ability does not necessarily applies to individual OTUs. In order to investigate this further we performed a differential abundance analysis.

### 3.2. Differential Abundance Along the Oxygen Gradient

The degree to which the abundance patterns of OTUs follow the oxygen gradient may be determined by differential abundance analysis. Notably, OTUs that exhibit only a weak or inconsistent response to the oxygen gradient should never be labelled as differentially abundant. If an OTU is never differentially abundant with regards to the oxygen gradient, then oxygen is probably not the optimal gradient along which to map its abundance and prevalence. Hence, differential abundance of individual OTUs may be used as a means of validating the classification results in the previous section. Here we show that such a lack of differential abundance is common within certain lineages and sketch explanations as to why this might be the case.

If two OTUs relate somewhat similarly and predictably to the oxygen concentration of their surroundings, we can expect that the ratio of abundance for these two OTUs will remain similar across samples with similar oxygen concentration when local variations for example in past depositional conditions cancel out across many cores, which is the case for our dataset. In other words: the variance of their ratios within this category of samples will be low. If, however, the relation changes as oxygen concentration decreases, we may expect that the variance in their ratio between two sample categories, e.g., high and low oxygen concentration, will be considerably higher than within each category; that they are differentially abundant with respect to each other between those sample categories. Since the above description translates to any gradient that covaries with oxygen, sample categories must be selected to be as meaningful to oxygen as possible in order to filter away effects from covariates like nitrate.

The differential abundance analysis quantifies the differential abundance for all OTU pairs and for all categories of samples (Figure 2C). The resulting metric, θ_*d*_, is a measure of the ratio between within- and between-category Variance of the Log-Ratio (Erb et al., 2017; Quinn et al., 2017). Therefore, the lower the θ_*d*_, the stronger we may expect at least one of the OTUs in the investigated pair to be associated with the oxygen concentration of their surroundings. We thus expect θ_*d*_ to decrease as the difference in oxygen concentration between the two investigated categories becomes larger (Figure 5). The ranking of sample-wise classification accuracy shown and discussed above (section 3.1) indicates that the 10-25 μM concentration interval constitutes a threshold across which the majority of OTUs should be differentially abundant. Indeed, more OTU pairs are differentially abundant between the very highest (cat. 1) and lowest (cat. 6), as well as anoxic (cat. 7) samples, and their θ_*d*_ is considerably lower than for other sample category pairs (Supplementary Figure S11). It should be noted that while certain OTUs apparently are not differentially abundant, this merely means that we cannot discard our null hypothesis that these OTUs are not differentially abundant along the oxygen gradient for this dataset and the settings of our analyses.

**Figure 6:**
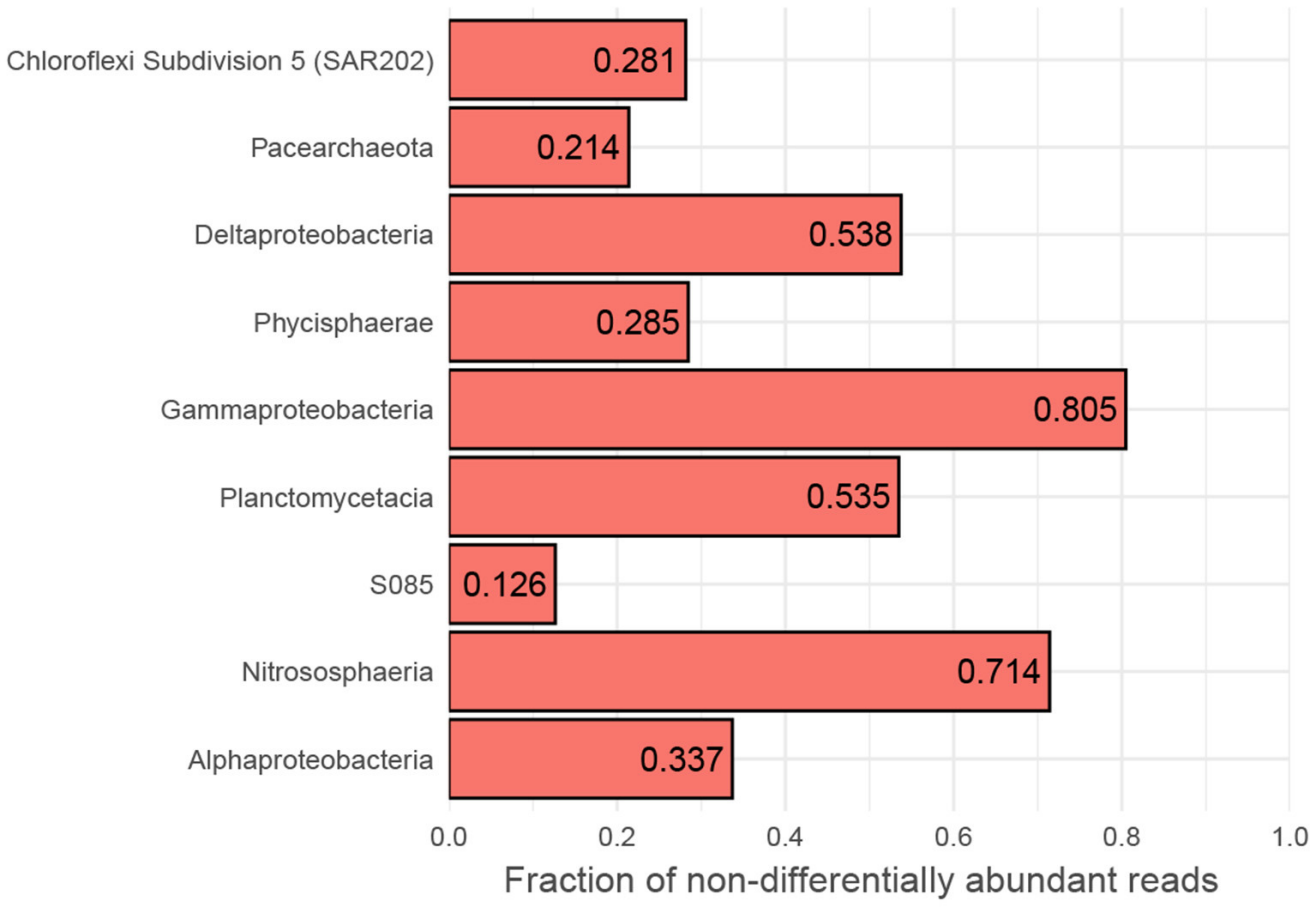
Fraction of non-responsive OTUs. Binning to class level of OTUs belonging to the 10 most abundant classes that are not differentially abundant between high- and low-oxic/anoxic (cat. 1 vs. 6 and 7) conditions in AMOR sediments. MD2896-B214 has no non-differentially abundant OTUs and is, therefore, not shown. A broad column suggests that OTUs accounting for most of the class's reads are too variable within oxygen categories compared to between to be labelled as differentially abundant.

**Figure 7:**
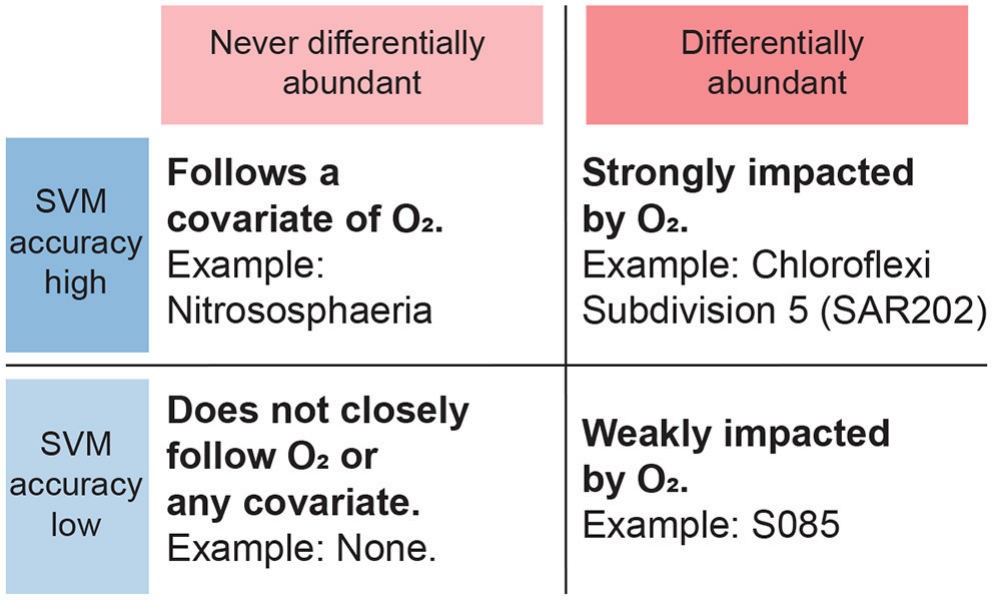
Interpretation of results from SVM and differential abundance analysis combined. Taxa that are never differentially abundant, corresponding to a wide bar on Figure 6, are taken to not primarily follow the oxygen gradient but instead possibly a corollary, regardless of SVM classification rates. Taxa that are differentially abundant with respect to the oxygen gradient and furthermore achieve high SVM classification rates, are expected to be strongly dependent on oxygen.

However, while the vast majority of OTUs exhibit differential abundance with respect to oxygen concentration changes along AMOR sediments at least once, 167 OTUs (3.8% of all) are never differentially abundant across either the 1–6 or 1–7 category pairs, corresponding to highest (>150 μM) vs. lowest oxygen concentration and anoxia (<10 μM), respectively. These OTUs are highly abundant and diverse, accounting for 41% of all reads and spanning 13 phyla and 32 classes. Nevertheless, certain classes are over-represented. Figure 6 shows the proportion of reads belonging to OTUs never labelled as differentially abundant for the 10 most abundant classes. Notably, the fractions of non-differentially abundant OTU reads affiliated with the highly abundant and prevalent Nitrososphaeria and Gammaproteobacteria are very high (Figure 6), as is the case for their respective order Nitrosopumilales and family Woeseiaceae (Supplementary Figure S12A,B). Figure 3 nevertheless shows that both classes have high classification rates. This is not a contradiction, but instead suggests that measurable oxygen may not be the primary explanatory variable for these classes (Figures 5, 7).

Woeseiaceae has been proposed as a core marine sediment microbiome member and commonly ranks among the most abundant families in reported studies where its presence could be taxonomically resolved (Bienhold et al., 2016; Mußmann et al., 2017). A recent review of its genomic repertoire indicated both aerobic and anaerobic growth, as well as widespread metabolic capabilities (Mußmann et al., 2017; Hoffmann et al., 2020). Our results supports a versatile lifestyle and suggest that the generalist role of Woeseiaceae renders oxygen concentration alone insufficient to explain their apparently very complex development in marine sediments and that more research is needed to elucidate their functional diversity at even lower taxonomic levels.

Focusing on Nitrososphaeria, 71.4% of all reads, and 24 of 169 OTUs, assigned to Nitrosopumilales were not differentially abundant. OTU_6, the most abundant OTU within the Nitrosophaeria class, exemplifies this well (Figures 5A,C): Its abundance decreases in anoxic samples, but it is still frequently too abundant to confidently say that it is differentially abundant with respect to the oxygen gradient. A blast alignment (Sina ACT) search (V4 region) against the SILVA 138 database (Pruesse et al., 2012) shows that all 24 OTUs not differentially abundant belong to the Nitrosopumilaceae family. All known representatives of this family are ammonia-oxidising archaea (AOA) converting ammonium to nitrite using oxygen (Könneke et al., 2005; Tully and Heidelberg, 2016; Orsi, 2018; Vuillemin et al., 2019), and with representatives with a very strong affinity and low threshold concentration for ammonium and oxygen (Martens-Habbena et al., 2009; Walker et al., 2010). However, we find that instead of oxygen, most reads ascribed to Nitrosopumilales (75.3%) are differentially abundant along the length of the nitrate gradient when performing the same differential abundance analysis against categories of nitrate concentration; see details in the Supplementary Methods. This finding could be due to the presence of oxygen below the detection limit of our measurement apparatus and rapid consumption of oxygen, but nevertheless underpins Nitrosopumilales' impact as nitrifiers along AMOR, and also shows that oxygen may not be the best variable to track presence and abundance for this order in deep-sea sediments. Alternatively, it could suggest that this group is able to oxidise ammonia under anoxic conditions, as recently shown for a member of this family, which is capable of producing its own oxygen (Kraft et al., 2022). The lack of differential abundance for many Nitrosopumilales representatives between oxygen-rich and anoxic sediments, and their presence in deep anoxic sediments (Kirkpatrick et al., 2019) as well as the aforementioned recent evidence of oxygen production in one representative (Kraft et al., 2022), furthermore underpins the complex relationship between this order and geochemical transition zones where the sources of required terminal electron acceptors may be hard to track.

High accuracy combined with a relatively low fraction of non-differentially abundant reads, conversely, indicates that oxygen concentration probably is the true primary explanatory variable (Figure 7). This applies for instance to the SAR202 clade under the Chloroflexi phylum, which is abundant in waters and sediments worldwide (Durbin and Teske, 2011; Mehrshad et al., 2018). Figure 5B shows the abundance of the most abundant SAR202 OTU, which is differentially abundant and exhibits a distinct drop in abundance as oxygen decreases below 25 μM. In an extensive study of the Chloroflexi phylum, Vuillemin et al. (2020) found that this clade was abundant in oxic sediments but quickly disappeared under anoxic conditions, in line with our findings. Through metagenomic and metatranscriptomic analyses, they furthermore revealed strong potential for SAR202 representatives to access recalcitrant organic matter, which may explain their abundance in oxic deep-sea sediments (Vuillemin et al., 2020).

## 4. Conclusions

In this study, we demonstrate the pronounced effect of oxygen on microbial community composition in Arctic deep-sea subsurface sediments. Using Support Vector Machines, we show that common microbial classes can be used to predict the oxygen concentration of their immediate environment with an accuracy far above that expected from random chance, thus quantifying the relationship between common microbial classes and the oxygen gradient in a manner that extends far beyond the mere oxic-anoxic binary. However, if this relationship is caused directly by the oxygen concentration or indirectly, e.g. through linkage to respiration rates as a function of degradable organic carbona availability, is unknown. We also show that a relatively homogeneous microbiome is recruited to the benthos. However, classification rate patterns show that the the composition of the most abundant classes then becomes more heterogeneous as oxygen drops below 25 μM, putting a biologically informed threshold for the onset of the oxic-anoxic transition zone. There is no significant correlation between relative abundance and achieved classification rate among the common taxa. Moreover, several highly abundant OTUs belonging to classes achieving high classification accuracy are not differentially abundant from high-oxic (>150 μM) to low-/anoxic (<10 μM) conditions. This suggests that the abundance and prevalence of highly abundant OTUs may be better explained by other variables than oxygen. For example, we find that the distribution of Nitrosopumilales is better explained by nitrate concentration, although possibly so because oxygen remains present but undetectable in deeper layers. The combined scale of our dataset and compositionally sound methodology used enable us to provide an enriched context for disentangling and interpreting the complex interplay between microbial communities and the oxygen gradient in deep-sea subsurface sediments. Our methods may be applied to any well-defined environmental gradient that follows a developing microbial community, regardless of ecosystem.

## Data Availability Statement

All fastq sequences required to build the dataset have been deposited to the NCBI SRA database and are available through project numbers PRJNA529480 and PRJNA789780. All other data sets may be found in the Supplementary Material online. The R code used to generate the analyses, results and figures presented in this study is available from www.github.com/tormolle/oxic_response.

## Author Contributions

SJ, SL, RZ, TB, DR, AD, IT, and RP collected and processed data. BH, SJ, and TM designed research, conducted statistical analyses, led the writing process, and wrote the first draft. TM, SJ, BH, and SB contributed actively to the analysis and writing process. All authors have read and approved the final manuscript.

## Funding

This research was funded by the Research Council of Norway (NFR) through the Centre for Geobiology (CGB) (project 179560), the KG Jebsen foundation, and the Mohn foundation through the TMS starting grant BFS2017REK03.

## Conflict of Interest

The authors declare that the research was conducted in the absence of any commercial or financial relationships that could be construed as a potential conflict of interest.

## Publisher's Note

All claims expressed in this article are solely those of the authors and do not necessarily represent those of their affiliated organizations, or those of the publisher, the editors and the reviewers. Any product that may be evaluated in this article, or claim that may be made by its manufacturer, is not guaranteed or endorsed by the publisher.
