# Supplementary material for: Mapping Microbial Abundance and Prevalence to Changing Oxygen Concentration in Deep-Sea Sediments Using Machine Learning and Differential Abundance
Source: Front Microbiol. 2022 May 18;13:804575. doi: 10.3389/fmicb.2022.804575 (PMC9158483; doi:10.3389/fmicb.2022.804575)
Supplement: Supplementary file 1 [file Data_Sheet_1.PDF]

Supplementary Material

1 SUPPLEMENTARY DATA

All the files listed below are available separately as part of the supplement.
all.otutab.nonchimeras.csv: Full, unprocessed OTU table.
arb-silva.de_align_resultlist_948641_nitrosopumilus.csv: Taxonomic assignment of Nitrosopumilales OTUs.
contaminant_genera.ods: List of known laboratory contaminants used for quality control in sequence post-processing.
mapping.ods: Mapping between sample id and depth used in data loading.
no3.xlsx: Nitrate measurements for all analysed cores.
otus.csv: Taxonomic assignment for all OTUs in the full, unprocessed OTU table.
oxygen_profiles.xlsx: Oxygen measurements for all analysed cores. **toc_profiles.xlsx:** Total Organic Carbon (TOC) measurements for all available cores.

2 SUPPLEMENTARY METHODS

2.1 Sample de-trending

In all reported cores, oxygen concentration decreases monotonically to depletion with depth along a well-defined diffusive gradient. To test whether sediment depth and oxygen concentration could therefore be confounded, we detrended OTU data sample-wise on first differences and analysed classified quaternary oxic state for comparison with non-detrended data. Detrending n samples by first differences results in $n - 1$ differences between samples. In order to ensure that the detrended dataset was of equal size as the non-detrended one, we included an additional anoxic sample between the preexisting three before detrending. The resulting comparison between detrended and non-detrended data is shown on Figure S13.

2.2 Overview of batches and samples

Table S1. Correspondence between sequencing batches, cores and number of samples, sampling horizons and number of blanks used in sequence processing.

Batch name	Cores (no. samples)	min-max sample depth [cm]	Blanks
CB03/06	GS10_PC12 (26),<br>GS13_CC02 (23),<br>GS14_GC02, (13),<br>GS14_GC14 (13)	206-1766<br>5-1880<br>10-215<br>1-200	1
CB08/05	GS14_GC04 (15),<br>GS14_GC08 (15),<br>GS14_GC09 (11),<br>GS14_GC12 (17),<br>GS15_GC01 (27)	5-230<br>10-340<br>10-190<br>10-357<br>5-390	3
CB10	GS16_GC04 (19)	2-300	1
CB11	GS16_GC05 (22),<br>GS16_GC06 (23),<br>GS16_GC07 (8)	5-350<br>5-350<br>5-80	2
CB14	GS17_GC02 (69),<br>GC17_GC05 (20)	5-240<br>5-400	3
CB15	GS17_GC04 (93)	3-290	5

2.3 Total Organic Carbon (TOC) measurements

Sediments were dried and TOC was measured on an element analyser (Analytikjena multi EA® 4000 (Jena, Germany) after removing inorganic carbon by addition of 1 mL of phosphoric acid.

2.4 Nitrate measurements and categorisation of nitrogenous sample categories

In brief, pore fluid measurements were taken using Rhizon samplers inserted into the sediments immediately after splitting shipboard. All sampling horizons can be found in the **no3.xlsx** supplementary file. Nutrients, nitrate among them, were measured shipboard colorimetrically using a Quaatro continuous flow analyzer (SEAL Analytical Ltd, Southampton, UK) according to the manufacturer's protocol. For further details, the reader is referred to the supplement of (Zhao et al., 2020).

As was the case with the oxic samples, most microbial samples did not have exactly corresponding pore fluid extractions and consequently nitrate measurements. We found that simple linear regression provided the best fit between measured and modelled values, cf. Figure S14. Based on visual inspection, any sample with an interpolated concentration <1 µM was set to 0. We then categorised all samples ($n = 184$) based on the interpolated nitrate concentrations. **Table S2** shows details of this categorisation.

3 SUPPLEMENTARY TABLES

Table S2. Category assignment, corresponding nitrate concentration intervals, and group size for samples

Category	nitrate [µM]	No. of samples
1	> 30	15
2	$30 - 25$	19
3	$25 - 20$	17
4	$20 - 15$	39
5	$15 - 10$	46
6	$10 - 5.0$	36
7	< 5.0	12

Table S3. Median accuracy and 95% confidence interval for quaternary classification among the ten most abundant microbial classes.

Class	Median	95% CI
Alphaproteobacteria	0.76	[0.63, 0.87]
Chloroflexi Subdivision 5 (SAR202)	0.76	[0.65, 0.85]
Deltaproteobacteria	0.76	[0.63, 0.85]
Phycisphaerae	0.74	[0.65, 0.85]
Nitrososphaeria	0.74	[0.61, 0.83]
Gammaproteobacteria	0.72	[0.59, 0.85]
Pacearchaeota	0.70	[0.63, 0.84]
Planctomycetacia	0.70	[0.59, 0.82]
S085	0.63	[0.48, 0.76]
MD2896-B214	0.54	[0.38, 0.67]

4 SUPPLEMENTARY FIGURES

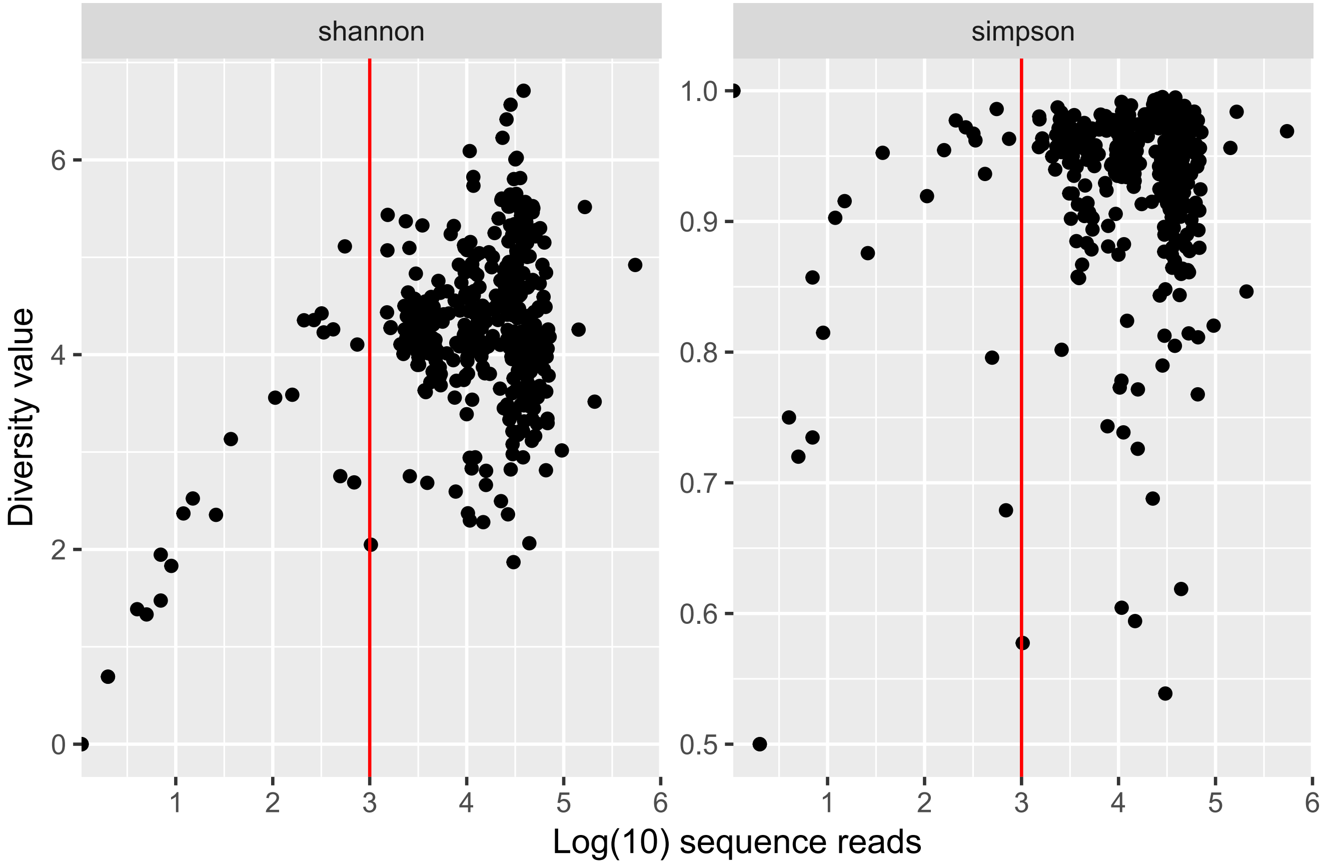

Figure S1. Diversity measurements versus library size. **A:** Shannon diversity. **B:** Simpson diversity.

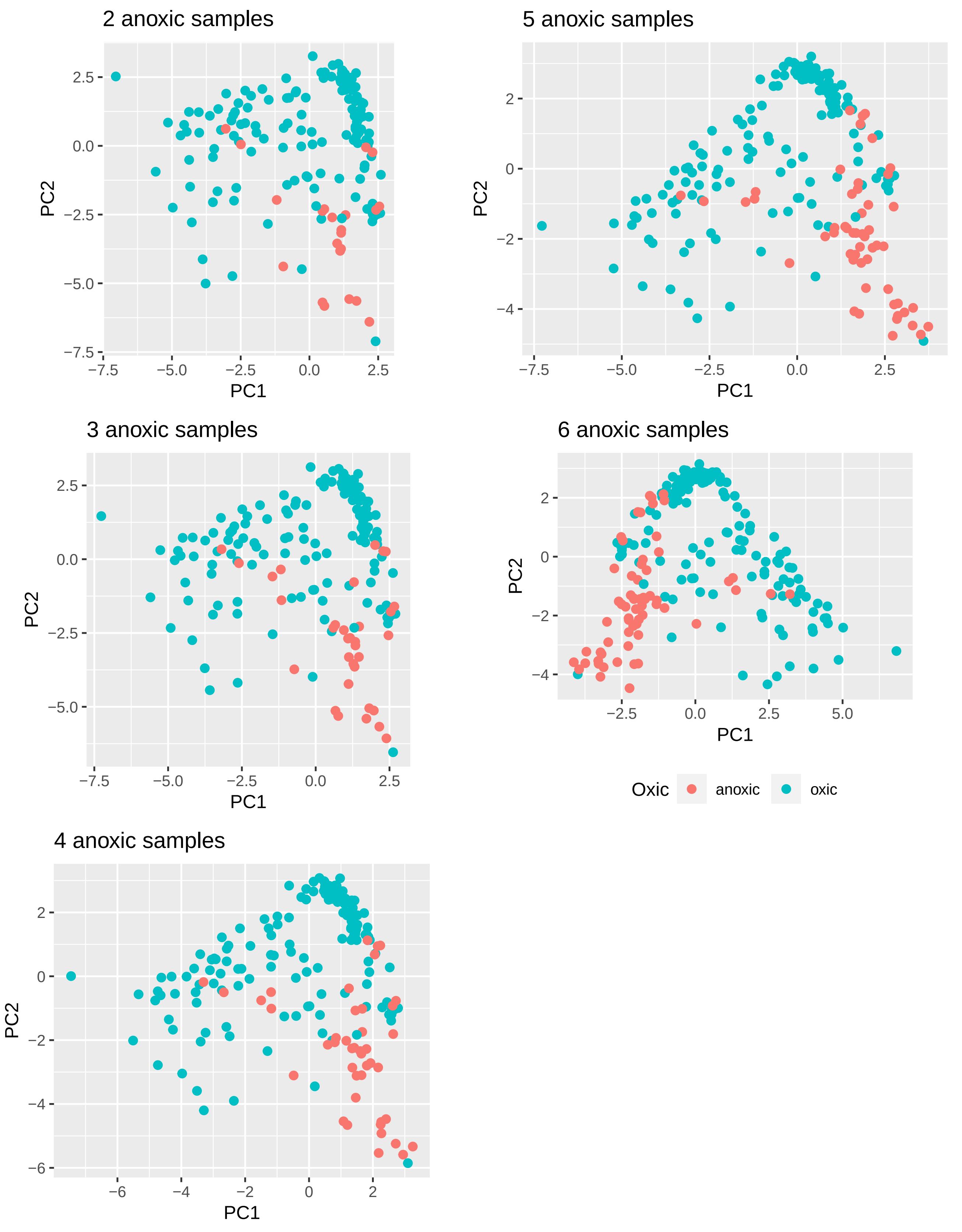

Figure S2. Sensitivity to number of anoxic samples. PCA ordination of zero-imputed, clr-transformed data with increasing numbers of anoxic samples (red) below the oxic segment (blue) for all 11 cores described.

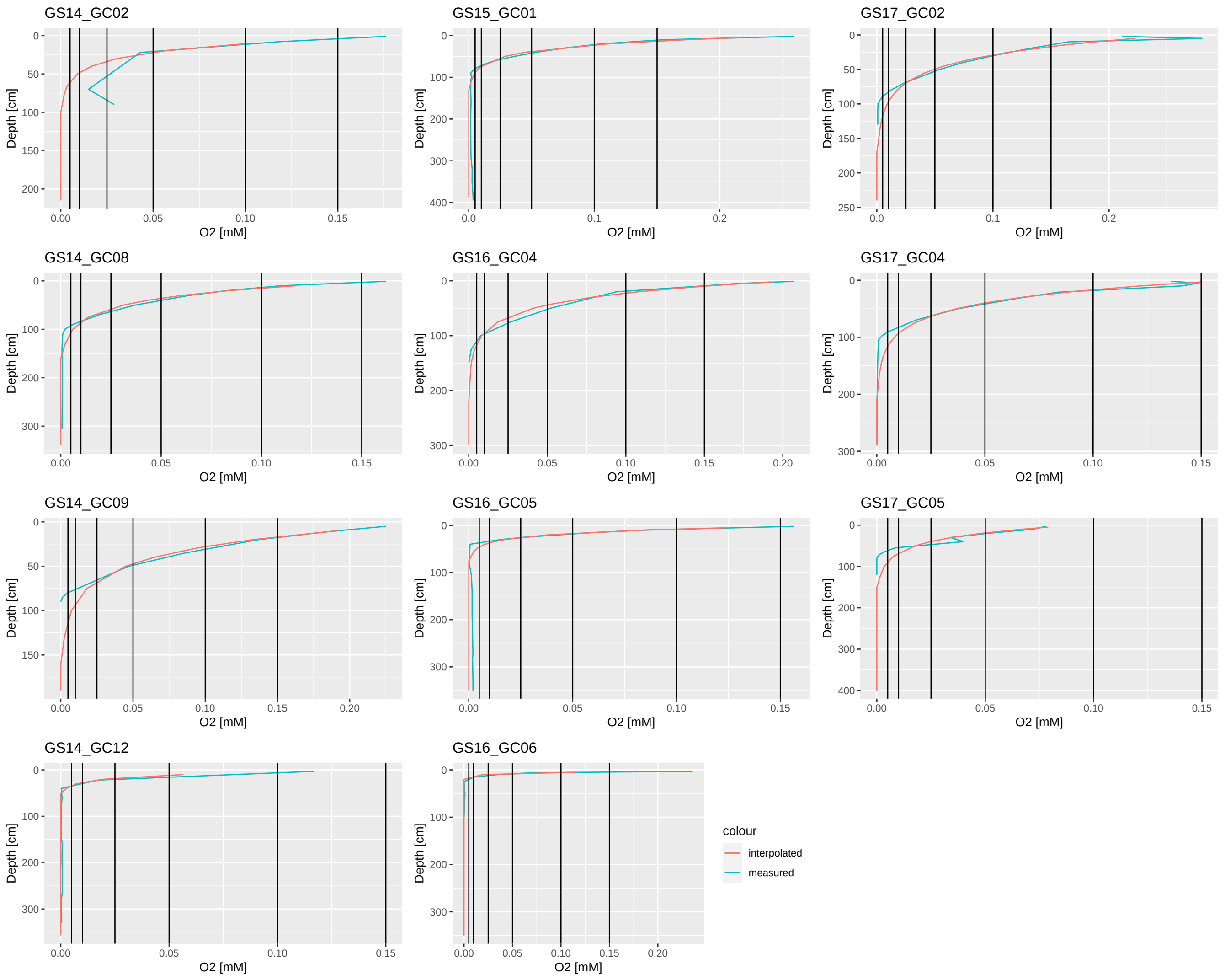

Figure S3. Measured and interpolated oxygen concentrations. We interpolated the concentration values for microbial horizons by fitting a non-linear least squares regression model with a mathematical expression for exponential decay: $C(d) = a \exp -bd$, where a and b are coefficients determined for each core and d is depth in cm. Vertical black lines denote divisions between oxygen categories, cf. Table 1 in the main text.

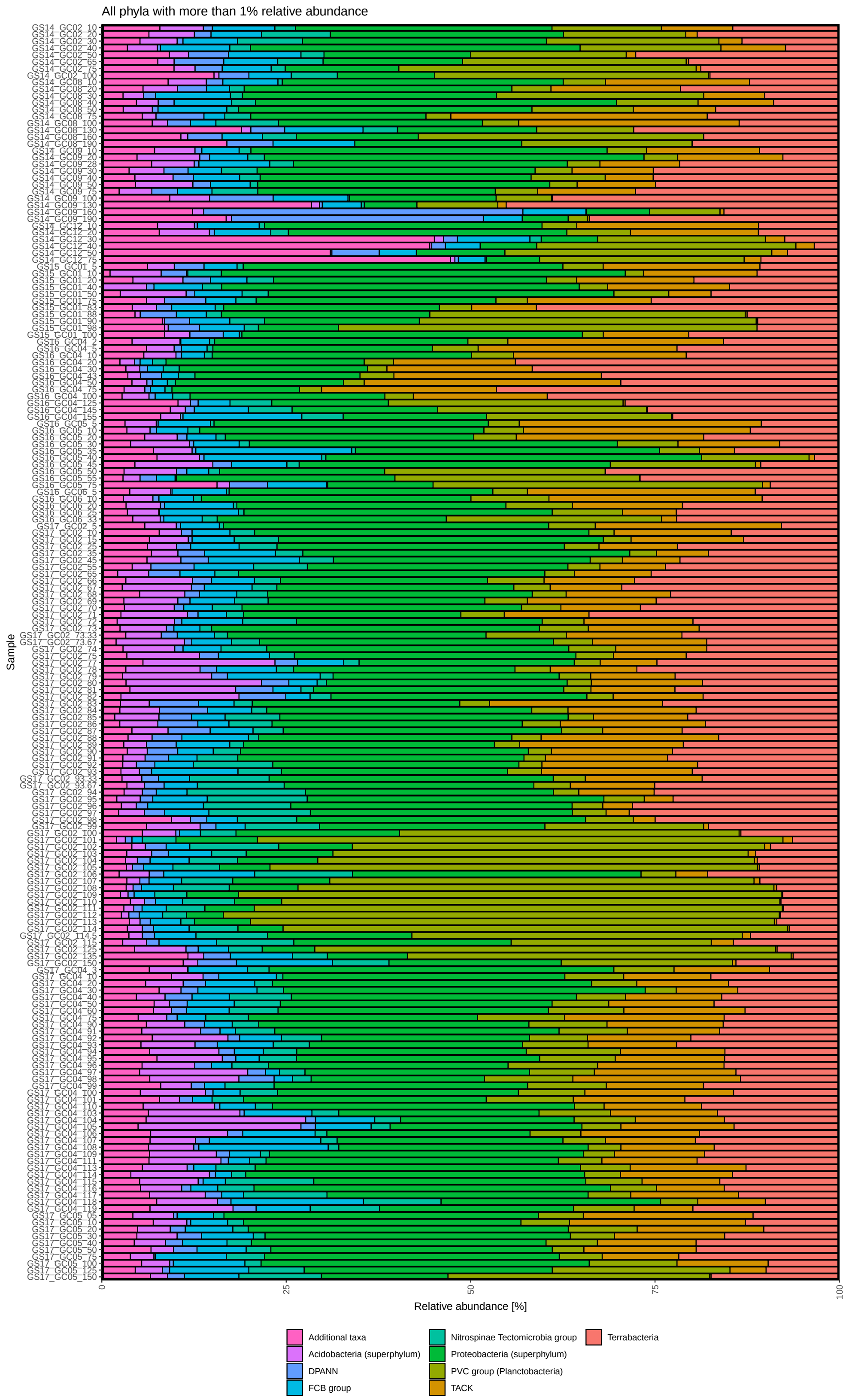

Figure S4. Taxonomic distribution of phyla. Only phyla with more than 1% relative abundance are shown. All other phyla are labelled as 'Additional taxa'.

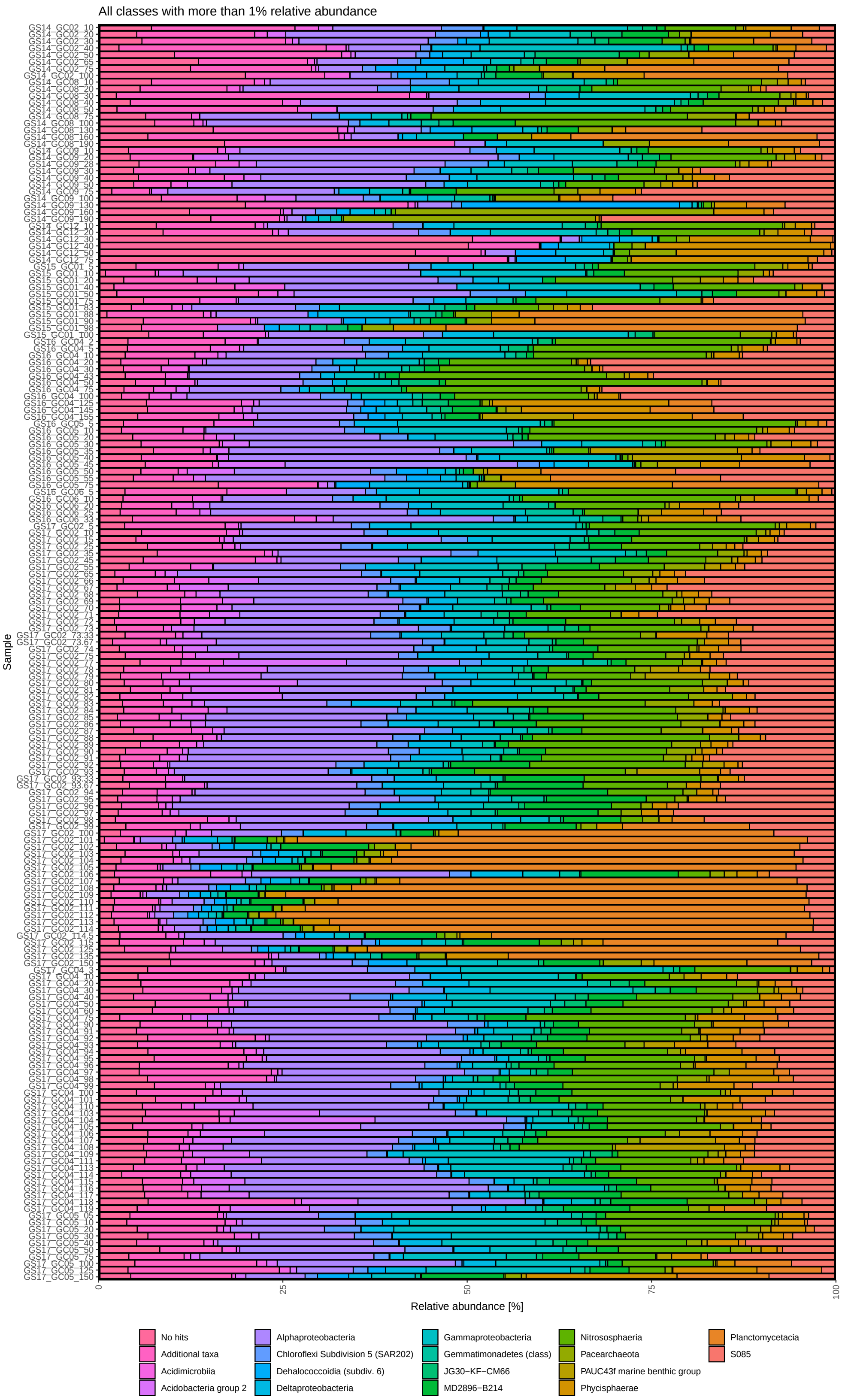

Figure S5. Taxonomic distribution of classes. Only classes with more than 1% relative abundance are shown. All other classes are labelled as 'Additional taxa'.

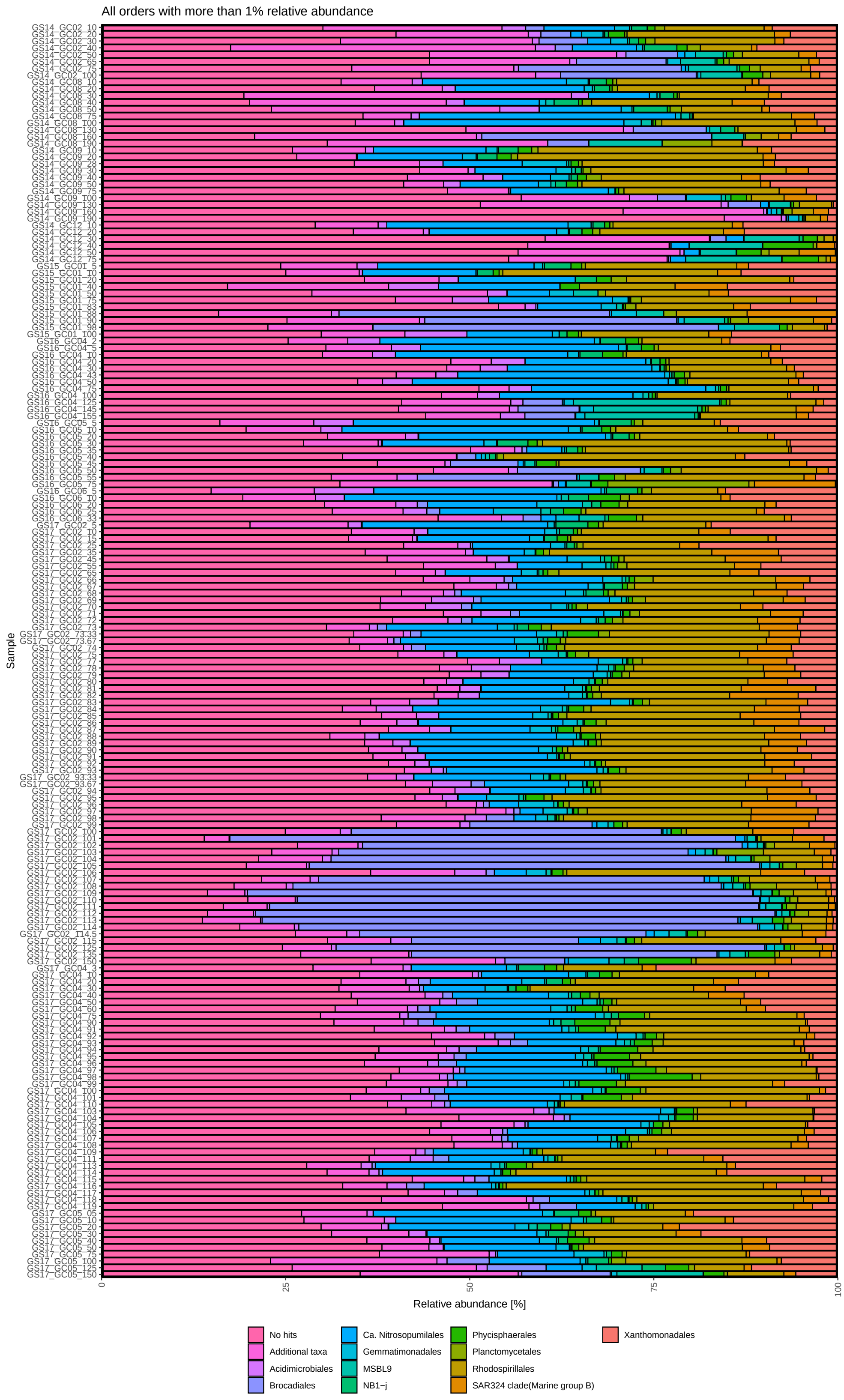

Figure S6. Taxonomic distribution of orders. Only orders with more than 1% relative abundance are shown. All other orders are labelled as ’Additional taxa‘.

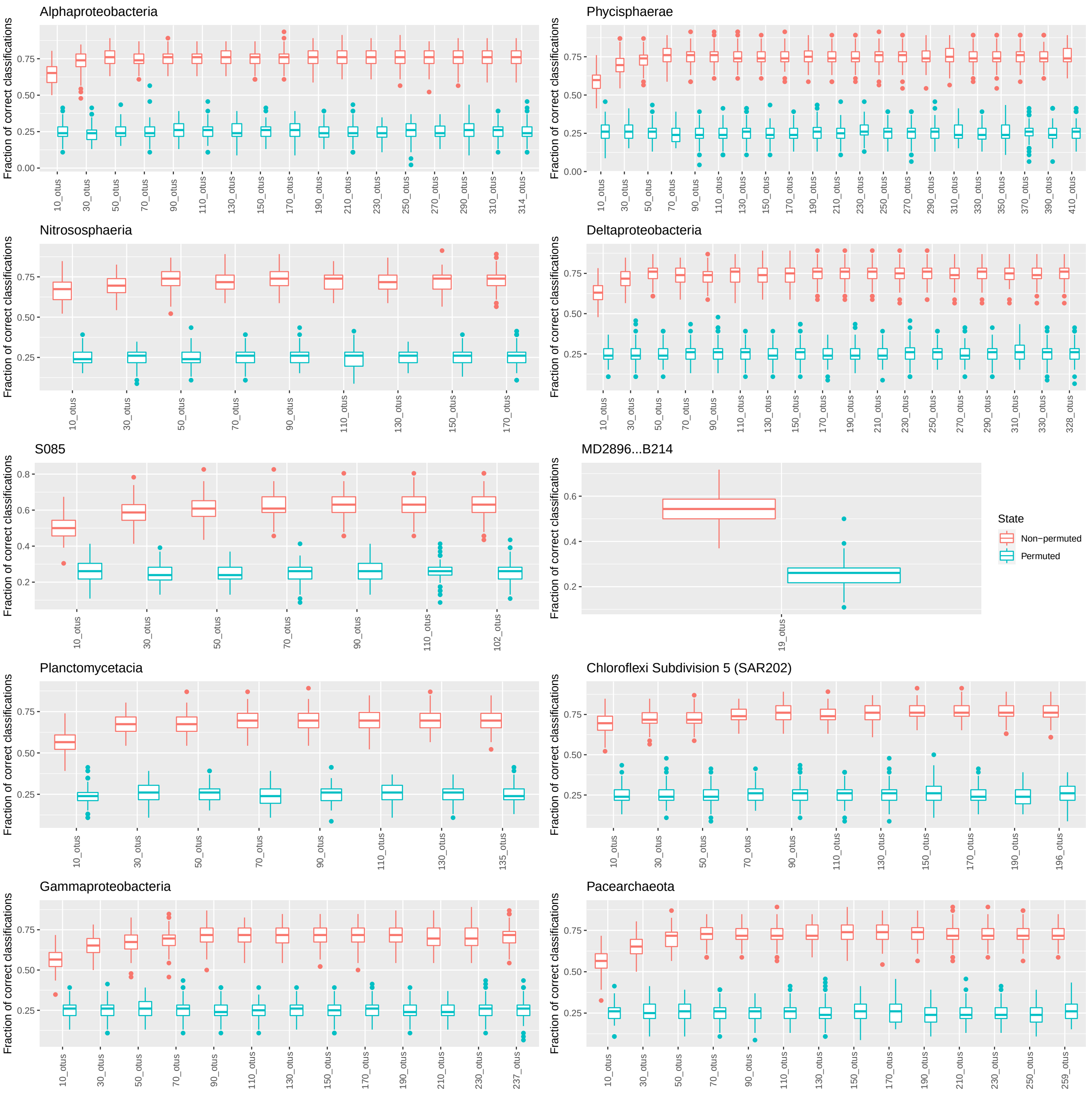

Figure S7. Classification rate as number of OTUs increases. Only results for the top 10 abundant classes are shown. Each box represents $n = 128$ iterations.

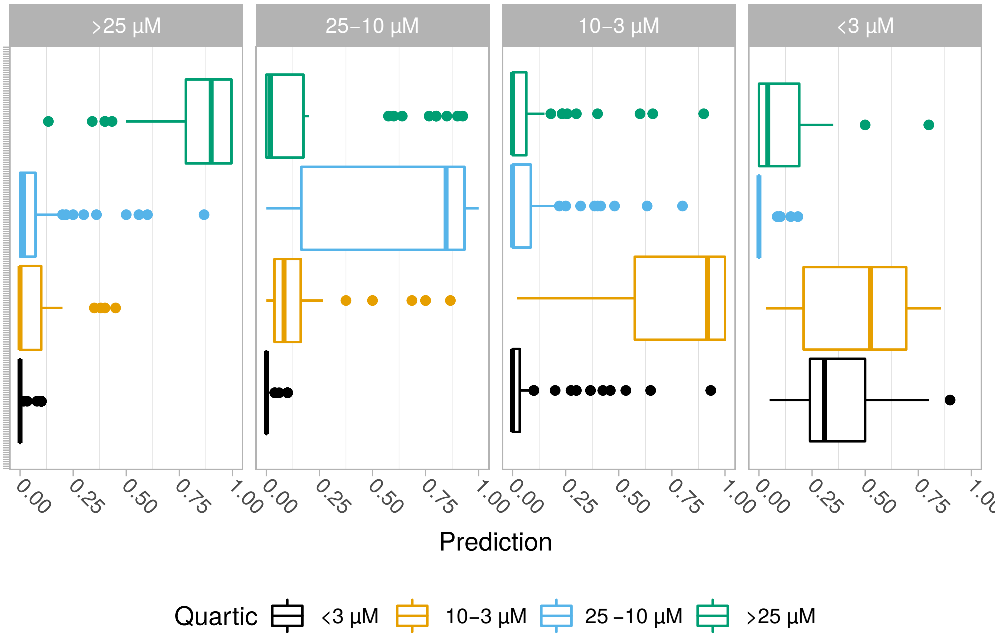

Figure S8. Quaternary classification rate with anoxia threshold lowered from 5 to 3 μM. In the case that the chosen threshold for labelling samples as anoxic at 5 μM, which is above the detection limit for the optode used on-board to measure concentration (3 μM), was causing high misclassification rate among anoxic samples, we tested lowering the threshold. Classification rate for anoxic samples did not improve.

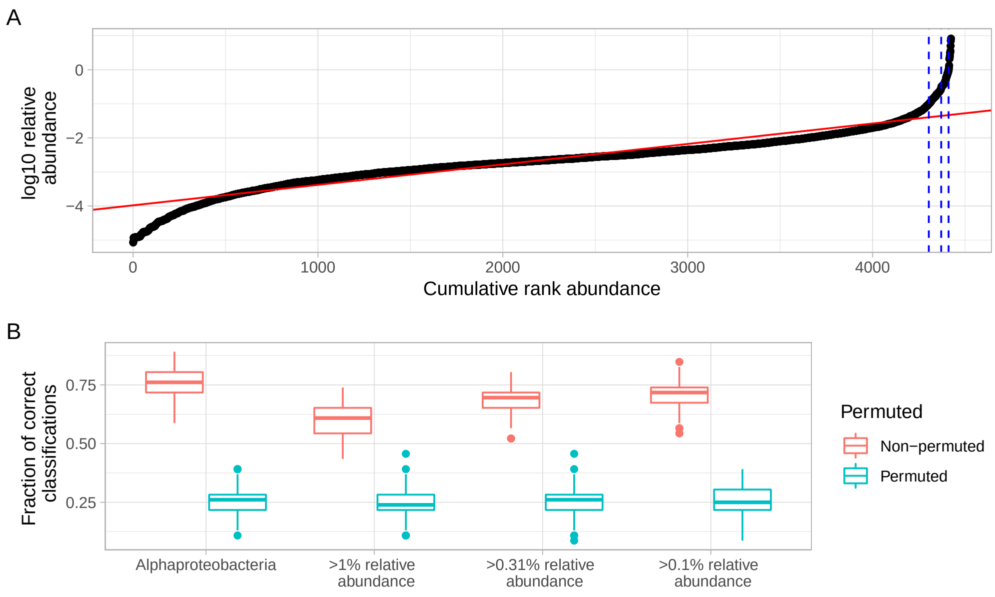

Figure S9. Accuracy of hyperdominant OTUs. **A:** The most abundant OTUs along AMOR belong to a category of representatives referred to as hyperdominant in ecological literature and are expected to be particularly good predictors of their environment ter Steege et al. (2013); de Vargas et al. (2015); Hannisdal et al. (2017). In order to test whether this was true for AMOR sediments, we selected three abundance thresholds (blue dashed lines: $> 1\%$, $> 0.31\%$ and $> 0.1\%$) and used all OTUs exceeding those as independent variables in quaternary classification. **B:** All subsets achieved significantly lower classification accuracy than Alphaproteobacteria, suggesting that the effect of hyperdominance is not pronounced in AMOR sediments.

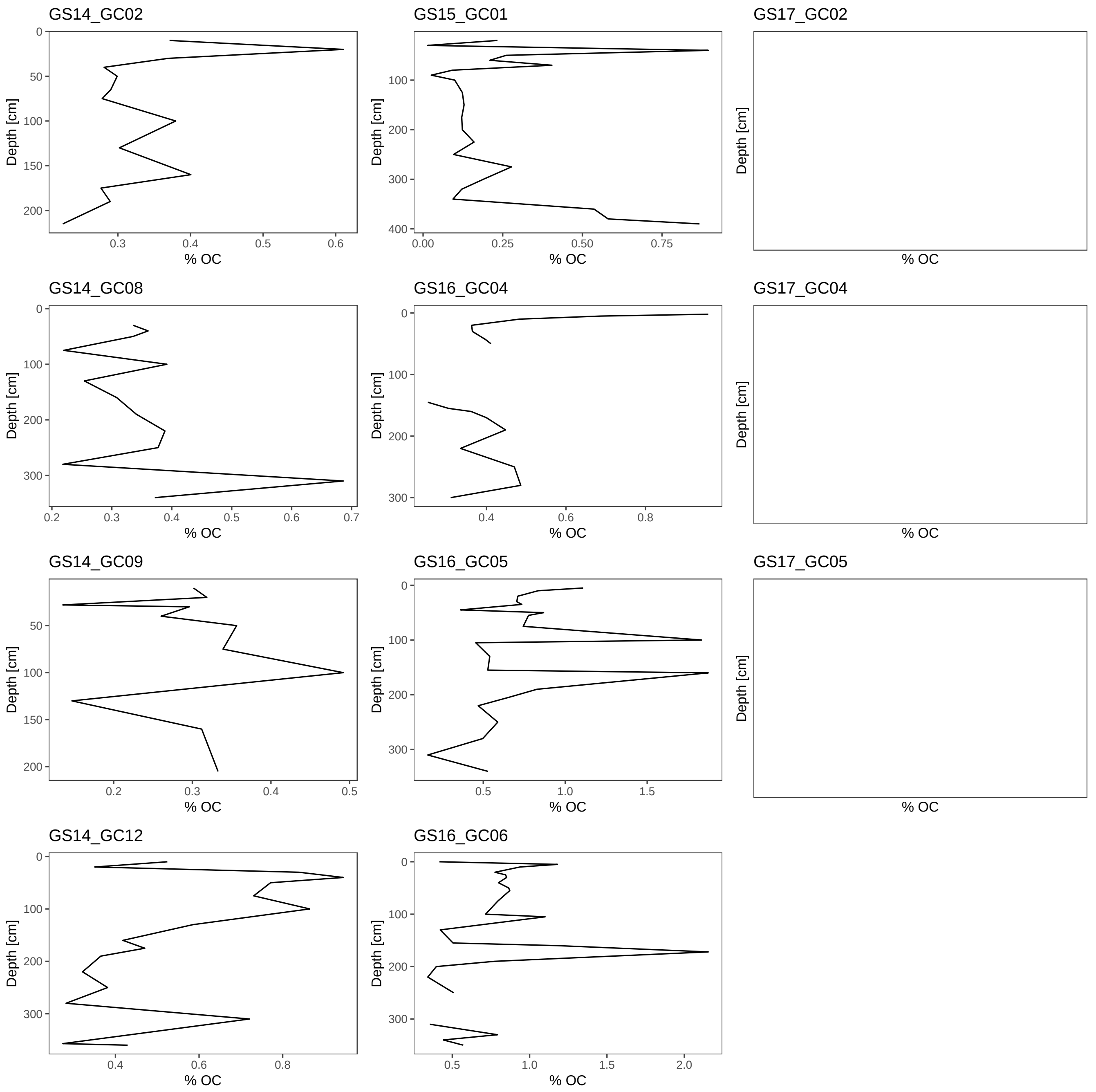

Figure S10. Total Organic Carbon (TOC) profiles. TOC only decreased significantly ($p < 0.05$) with depth in GS14_GC12, GS16_GC04 and GS16_GC05. For all other cores, correlations were insignificantly negative or positive.

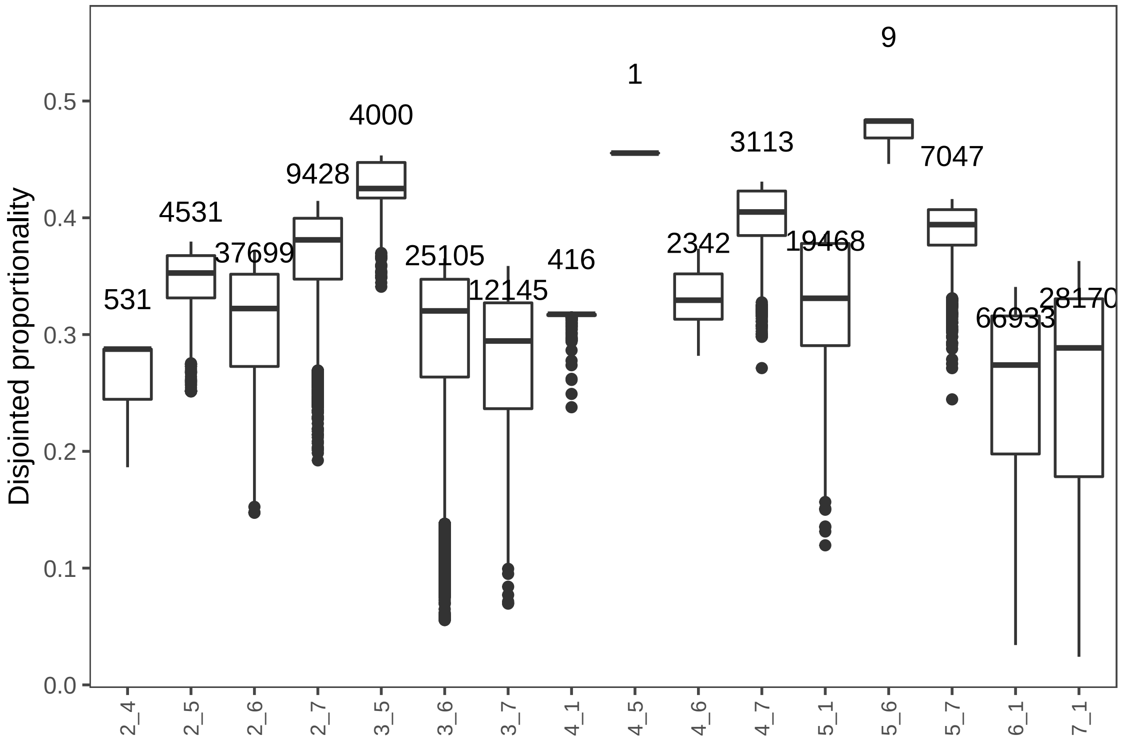

Figure S11. Number of differentially expressed OTU pairs. Boxes display the range of disjointed proportionality (θ_d) for each category pair (cf. Table 1 in the main manuscript). The number atop each column denotes the number of OTU pairs in which at least one OTU is differentially expressed in relation to the other after requiring θ_d <0.50 and retaining only the 25% lowest values for each category pair.

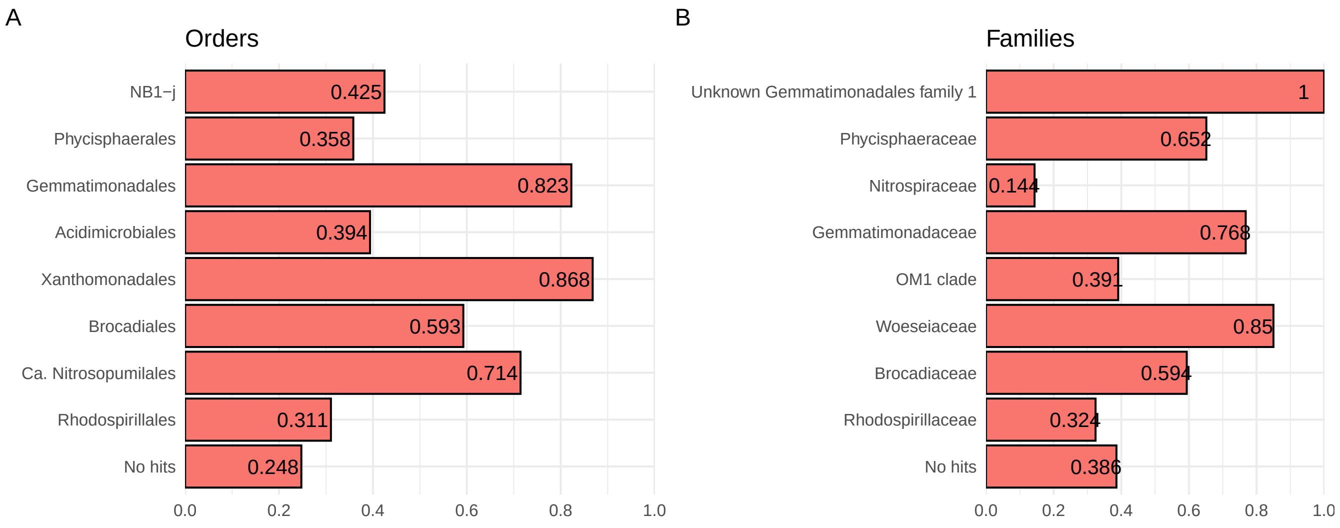

Figure S12. Orders and families not differentially expressed along the oxygen concentration. OTUs that were never differentially expressed once when comparing categories 1-6 and 1-7, binned to **A:** order, and **B:** family level.

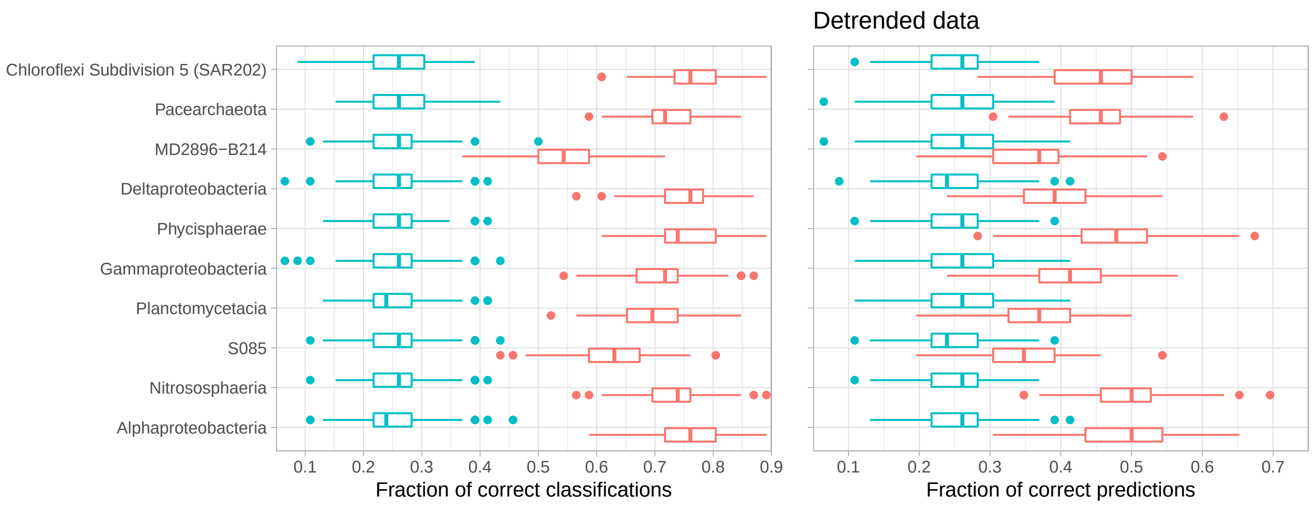

Figure S13. Comparison of classification rates for detrended and non-detrended OTU data. **A:** Figure 3 as it appears in the main manuscript. **B:** Identical analysis performed on detrended data. Detrending was done core-wise on first differences between samples and reveal a still-present and significant signal from oxygen visible as classification rates significantly above random chance among all 10 examined classes.

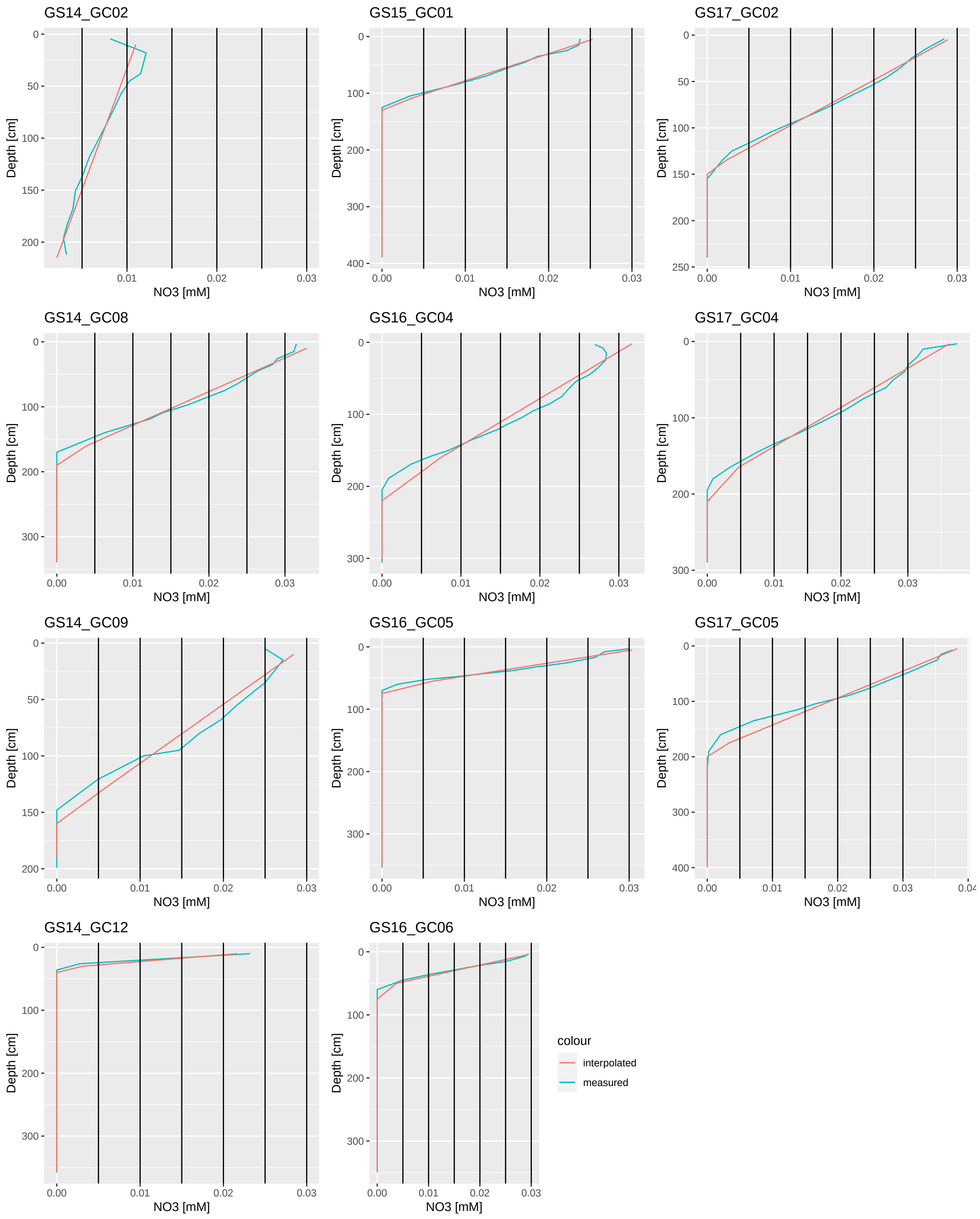

Figure S14. Measured and interpolated nitrate profiles. We used linear regression to interpolate measured nitrate profiles. We included all non-zero measurements plus the first zero to obtain the linear fit.

REFERENCES

de Vargas, C., Audic, S., Henry, N., Decelle, J., Mahé, F., Logares, R., et al. (2015). Eukaryotic plankton diversity in the sunlit ocean. *Science* 348. doi:10.1126/science.1261605

Hannisdal, B., Haaga, K. A., Reitan, T., Diego, D., and Liow, L. H. (2017). Common species link global ecosystems to climate change: dynamical evidence in the planktonic fossil record. *Proceedings of the Royal Society B: Biological Sciences* 284, 20170722. doi:10.1098/rspb.2017.0722

ter Steege, H., Pitman, N. C. A., Sabatier, D., Baraloto, C., Salomão, R. P., Guevara, J. E., et al. (2013). Hyperdominance in the amazonian tree flora. *Science* 342. doi:10.1126/science.1243092

Zhao, R., Mogollón, J. M., Abby, S. S., Schleper, C., Biddle, J. F., Roerdink, D. L., et al. (2020). Geochemical transition zone powering microbial growth in subsurface sediments. *Proceedings of the National Academy of Sciences* 117, 32617–32626. doi:10.1073/pnas.2005917117
